# Pan-genome analyses of model fungal species

**DOI:** 10.1099/mgen.0.000243

**Published:** 2019-02-04

**Authors:** Charley G. P. McCarthy, David A. Fitzpatrick

**Affiliations:** ^1^​ Genome Evolution Laboratory, Department of Biology, Maynooth University, Maynooth, Co. Kildare, Ireland; ^2^​ Human Health Research Institute, Maynooth University, Maynooth, Co. Kildare, Ireland

**Keywords:** fungal pangenomes, comparative genomics, yeast, *Aspergillus*, *Cryptococcus*

## Abstract

The concept of the species ‘pan-genome’, the union of ‘core’ conserved genes and all ‘accessory’ non-conserved genes across all strains of a species, was first proposed in prokaryotes to account for intraspecific variability. Species pan-genomes have been extensively studied in prokaryotes, but evidence of species pan-genomes has also been demonstrated in eukaryotes such as plants and fungi. Using a previously published methodology based on sequence homology and conserved microsynteny, in addition to bespoke pipelines, we have investigated the pan-genomes of four model fungal species: *Saccharomyces cerevisiae*, *Candida albicans*, *Cryptococcus neoformans* var. *grubii* and *Aspergillus fumigatus*. Between 80 and 90 % of gene models per strain in each of these species are core genes that are highly conserved across all strains of that species, many of which are involved in housekeeping and conserved survival processes. In many of these species, the remaining ‘accessory’ gene models are clustered within subterminal regions and may be involved in pathogenesis and antimicrobial resistance. Analysis of the ancestry of species core and accessory genomes suggests that fungal pan-genomes evolve by strain-level innovations such as gene duplication as opposed to wide-scale horizontal gene transfer. Our findings lend further supporting evidence to the existence of species pan-genomes in eukaryote taxa.

## Data Summary

All the genomic sequence data has been previously uploaded to the National Center for Biotechnology Information (NCBI) GenBank, and links to relevant articles or NCBI BioProject pages are included in Table S1 (available with the online version of this article). Gene model prediction and post-processing pan-genome analysis pipelines are available from https://github.com/chmccarthy/pangenome-pipelines.

**Impact Statement**

Recent prokaryotic genomic studies of multiple individuals from the same species has uncovered large differences in the gene content between individuals. It has become increasingly common to refer to species with multiple genomes sequenced in terms of their ‘pan-genome’. The pan-genome is the union of ‘core’ conserved genes and all ‘accessory’ non-conserved genes across all strains of a species. Species pan-genomes have been analysed in many prokaryotic species, but have been recently demonstrated in eukaryotes such as plants and fungi as well. Here, we have investigated the pan-genomes of four model fungal species namely, *Saccharomyces cerevisiae*, *Candida albicans*, *Cryptococcus neoformans* var. *grubii* and *Aspergillus fumigatus*. Each species is a model organism for fungal evolutionary biology, genomics and comparative genomics. Our results show that between 80 and 90 % of gene models per strain are core genes that are highly conserved, many of which are involved in housekeeping and conserved survival processes. The remaining accessory gene models are clustered within subterminal regions, and may be involved in pathogenesis and antimicrobial resistance. Analysis of the ancestry of species core and accessory genomes suggests that fungal pan-genomes evolve by strain-level innovations such as gene duplication as opposed to wide-scale horizontal gene transfer. Our findings lend further supporting evidence to the existence of species pan-genomes in eukaryote taxa.

## Introduction

**Fig. 1.**
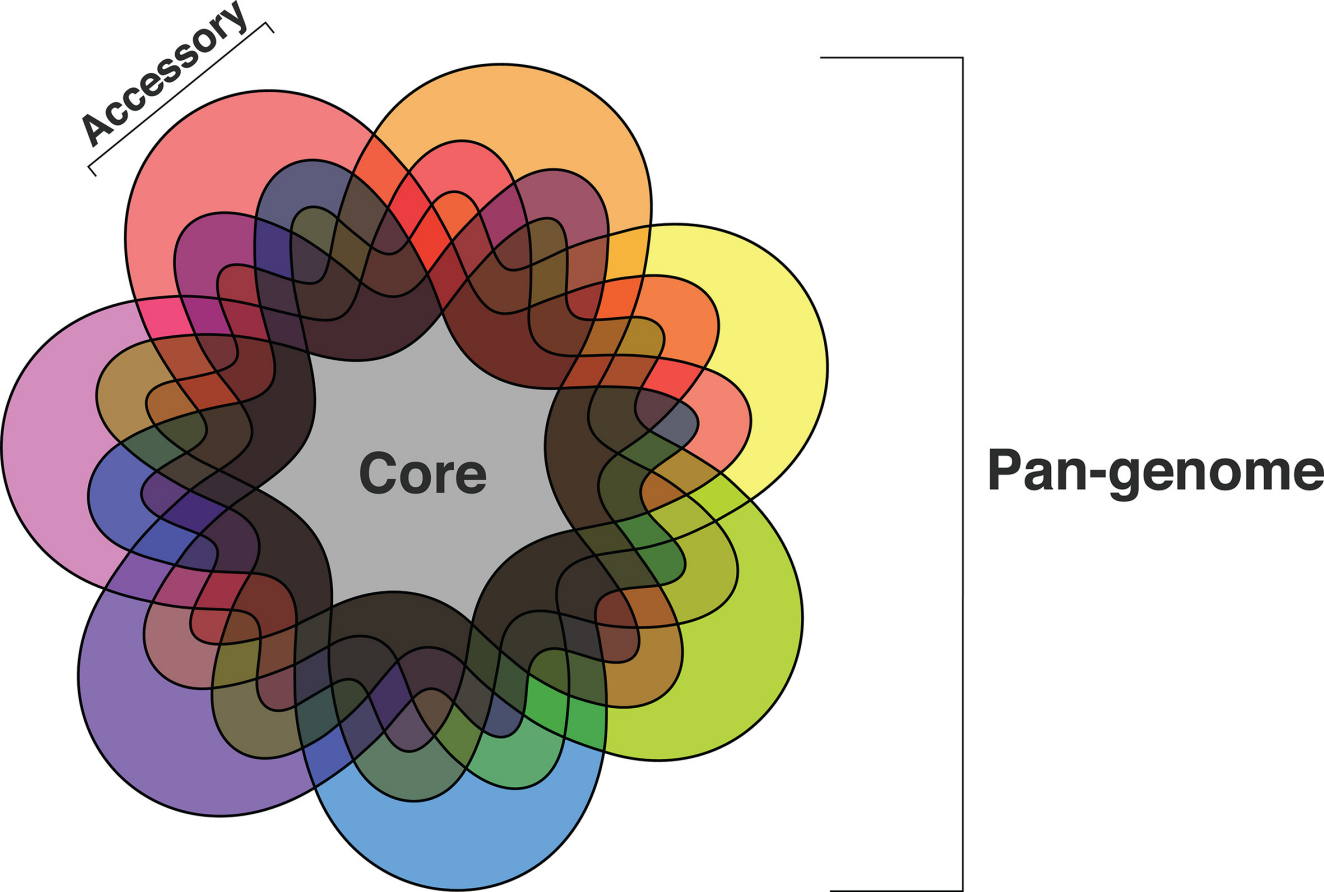
Seven-set Venn diagram representing a hypothetical species pan-genome. Each set represents genes/gene models conserved across strains of a given species. The core species genome (grey) is defined as the set of all genes/gene models conserved across all strains of a species, while the accessory genome consists of all genes/gene models not universally conserved within a species.

Many fields of eukaryote functional and comparative genomics rely on the use of curated reference genomes intended to be broadly representative of a given species. Regardless of their quality, reference genomes do not and cannot contain all genetic information for a species due to genetic and genomic variation between individuals within a species [1]. To account for such variation, it has become increasingly common to refer to species with multiple genomes sequenced in terms of their ‘pan-genome’, which is defined as the union of all genes observed across all isolates/strains of a species (Fig. 1). The pan-genome of a species is then usually subdivided into two components. (i) The ‘core’ genome, containing genes conserved across all observed genomes from a species. These genes are usually, but not always, essential for the viability of an individual organism [2]. (ii) The ‘accessory’ or ‘dispensable’ genome, containing genes specific to sets of isolate genomes or individual isolate genomes within a species. These genes could influence phenotypic differences between isolates; for example, in bacteria, antibiotic-resistant and antibiotic-susceptible isolates of the same species may have different accessory genomes [2].

A species’ pan-genome can evolve as a consequence of lifestyle: sympatric species may have large pan-genomes (and thus a large degree of intraspecific variation), while environmentally isolated or highly specialized species have smaller pan-genomes [2–5]. The existence of a species pan-genome in prokaryotes was first demonstrated across eight pathogenic strains of *Streptococcus agalactiae* in 2005 [6], and was quickly confirmed by similar analysis of exemplar bacteria and archaea, including *Haemophilus influenzae*, *Escherichia coli* and *Sulfolobus islandicus* [7–11]. Over 40 prokaryote species had their pan-genomes described in the literature by 2013 [2]. Many tools for pan-genome analysis have been published in recent years, which utilize methods such as whole-genome alignment, read mapping, clustering algorithms or de Bruijn graph construction [12–16].

Although the concept of the species pan-genome is well-established in comparative prokaryote genomics, it has only recently been extended to comparative intraspecific studies of eukaryotes. This is despite repeated observation of intraspecific genomic content variation in eukaryotes dating back to the first intraspecific comparative analyses of *Saccharomyces cerevisiae* genomes in the mid-2000s [17–20]. The relative dearth of eukaryotic pan-genome analysis in the literature is due in part to the relative difficulty of sequencing and analysing large eukaryotic genome datasets relative to prokaryotes [21]. Additionally, while horizontal gene transfer (HGT) is thought to be the driving influence in prokaryotic gene family and pan-genome evolution, HGT occurs in far lower rates in eukaryotes and is more difficult to detect [22–26]. Despite these challenges, there have been a number of recent studies of intraspecific variation within diverse eukaryote taxa that show strong evidence for the existence of a eukaryotic pan-genome in some form. For example, comparative analysis of nine diverse cultivars of *Brassica oleracea* found that ~19 % of all genes analysed were part of the *B. oleracea* accessory genome, with ~2 % of these being cultivar-specific [27]. A similar comparison of seven geographically diverse wild soybean (*Glycine soja*) strains found approximately the same 80 : 20 proportion of core to accessory gene content within the wild soybean pan-genome, while larger accessory genome sizes have been reported in wheat, maize, grasses and *Medicago* [28–32]. Individual strains of the coccolitophore *Emiliania huxleyi* have an accessory complement of up to 30 % of their total gene content, which varies with geographical location [33]. In fungi, a number of studies of the *Saccharomyces cerevisiae* pan-genome, including a recent large-scale analysis of genome evolution across 1011 strains, have shown evidence for an accessory genome of varying size, as well as large variation in subterminal regions across multiple *Saccharomyces cerevisiae* strains [13, 34–36], and recent analysis of the *Zymoseptoria tritici* pan-genome found that up to 40 % of genes in the total *Z. tritici* pan-genome were either lineage or strain-specific [37].

The methods of pan-genome evolution within eukaryotes in the absence of rampant HGT appears to vary among species, and can include genome rearrangement events or more discrete adaptive evolution processes. In plants, accessory genomes may evolve as a result of varying levels of ploidy, heterozygosity and whole-genome duplication within species, as well as adaptive changes and the evolution of phenotypic differences, such as in *B. oleracea* [27]. Adaptive evolution has also influenced the evolution of the *Emiliania huxleyi* pan-genome, with strains containing varying amounts of nutrient acquisition and metabolism as a result of niche specialization [33]. High levels of functionally redundant accessory genome content can be observed within the *Z. tritici* species pan-genome, which is thought to arise from the species’ own genome defence mechanisms inducing polymorphisms as opposed to gene duplication events [37]. Peter *et al.* [36] observed a large proportion of accessory genes within *Saccharomyces cerevisiae* appear to have arisen via introgression from closely related *Saccharomyces* species, with a smaller number originating from HGT events with other yeasts [36].

In this study, we have adapted a method of prokaryotic pan-genome analysis that identifies putative pan-genomic structure within species by accounting for conserved genomic neighbourhoods (CGNs) between strain genomes and applied it to eukaryote analysis [38] (Fig. S1). We have used this method in tandem with bespoke pre- and post-processing pipelines that analyse the extent of gene duplication within species pan-genomes (available from https://github.com/chmccarthy/pangenome-pipelines) to construct and characterize the pan-genomes of four exemplar fungal species: *Saccharomyces cerevisiae*, *Candida albicans*, *Cryptococcus neoformans* var. *grubii* and *Aspergillus fumigatus*. All four species are model organisms in eukaryotic genomics and play important roles in human health and lifestyles; *Saccharomyces cerevisiae* is used extensively in biotechnology, *Candida albicans* is an opportunistic invasive pathogen and the second-most common cause of fungal infection, *Cryptococcus neoformans* var. *grubii* is an intracellular pathogen that causes meningitis in immunocompromised hosts, and *Aspergillus fumigatus* is an opportunistic respiratory pathogen [39–43]. We have found strong evidence for pan-genomic structure within all four fungal species. In line with previous analyses of other eukaryotes, we found that approximately 80–90 % of fungal species’ pan-genomes are composed of core genes, while the remainder is composed of strain or lineage-specific accessory genes. Analysis of the origin of fungal pan-genomes suggests that fungal accessory genomes are enriched for genes of eukaryotic origin and arise via eukaryotic innovations such as gene duplication as opposed to large-scale HGT. Functionally, fungal core genomes are enriched for both housekeeping processes and essential survival processes in pathogenic species, whereas many fungal accessory gene models are found within clusters in the terminal and subterminal regions of genomes and are enriched for processes that may be implicated in fungal pathogenicity or antimicrobial resistance. Our findings complement the increasing amount of studies showing evidence for pan-genomic structure in eukaryote species.

## Methods

### Dataset assembly

For each of the four fungal species chosen, we obtained strain genome assemblies from the National Center for Biotechnology Information's (NCBI’s) GenBank facility (Table S1). Strains were selected based on geographical and environmental diversity where possible (Table S1). The predicted protein set from each species’ reference genome was also obtained from GenBank. For each strain genome in each species dataset, translated gene model and gene model location prediction was performed using a bespoke prediction pipeline consisting of three parts (Fig. S2).

(i) Reference proteins were queried against individual strain genomes using Exonerate with a heuristic protein2genome search model [44]. Translated gene model top hits whose sequence length was ≥50 % of the query reference protein’s sequence length were considered homologues and included in the strain gene model set. The genomic locations of these gene models were included in the strain genomic locations dataset.

(ii) *Ab initio* hidden Markov model-dependent gene model prediction was carried out using GeneMark-ES, with self-training and a fungal-specific branch point site prediction model enabled [45]. Predicted gene models whose genomic locations did not overlap with any gene models previously predicted via the first step were included in the strain gene model set. The genomic locations of these gene model were also included in the strain genomic locations dataset.

(iii) Finally, position weight matrix-dependent gene model prediction was carried out for all remaining non-coding regions of the genome using TransDecoder [46]. For *Saccharomyces cerevisiae* and *Candida albicans* strain genomes, these gene models were additionally screened against a dataset of known ‘dubious’ pseudogenes in each species taken from their respective public repositories using blastp with an *E* value cut-off of 10^−4^ [47, 48]. Predicted gene models whose top blastp hit against a known dubious pseudogene had a sequence coverage of ≥70 % were removed from further processing. All remaining predicted gene models with a length of ≥200 aa and a coding potential score of 100 or greater as assigned by TransDecoder were included in the final strain gene model set. Their corresponding genomic locations were also included in the strain genomic locations dataset.

Thus, for each strain genome in a species dataset, a gene model set and corresponding genomic location set was constructed using two initial independent prediction methods; a search for gene models orthologous to the reference protein set and an *ab initio* prediction approach, followed by a ‘last resort’ approach for predicting gene models in genomic regions for which gene models had not been previously called. We used this approach to ensure consistency in gene models calls between strains and to reduce the potential of poor heterogenous gene model calling within each species dataset, which would in turn reduce the number of false positives/negatives in our analysis. The completeness of each set of predicted gene models was assessed using BUSCO with the appropriate BUSCO dataset for each species [49] (Table S1). For each species dataset, all strain genome gene model sets were combined and an all-vs-all blastp search was carried out for all predicted gene models using an *E* value cut-off of 10^−4^. The results of the blastp search were used as input for PanOCT along with the combined genomic location data for each strain genome in a species dataset [38]. Further information for each species dataset is detailed below.

### Saccharomyces cerevisiae

Genomic data for 100 *Saccharomyces cerevisiae* strains were obtained from the NCBI’s GenBank facility. Of these 100 genomes, 99 had previously been included in the geographically and phenotypically diverse ‘100-genomes strains’ (100GS) resource for *Saccharomyces cerevisiae* [50]. For our analysis, we excluded the 100GS European vineyard strain M22 as its lower assembly quality prevented us from carrying out *ab initio* gene model prediction using GeneMark-ES [45, 50]. In its place, we included the European commercial winemaking strain Lalvin EC118 [51]. The protein set for the reference *Saccharomyces cerevisiae* strain S288C was also obtained from GenBank [40]. Construction of the *Saccharomyces cerevisiae* pan-genome dataset was performed as detailed above, with potentially dubious gene model predictions for each strain genome checked against a dataset of 689 known dubious *Saccharomyces cerevisiae* gene models obtained from the *Saccharomyces* Genome Database (SGD) [17]. The completeness of each strain’s gene model dataset was assessed using 1711 *Saccharomyces cerevisiae* BUSCOs from the Saccharomycetales dataset; on average ~1677 BUSCOs (~98 %) were retrieved as complete gene models in each strain (Table S1). In total, 575 940 gene models and corresponding unique genomic locations were predicted for 100 *Saccharomyces cerevisiae* genomes (Table S1).

### Candida albicans

Genomic data for 34 *Candida albicans* strains were obtained from the NCBI’s GenBank facility, encompassing predominantly clinical or presumed-clinical strains isolated from North America, Europe and the Middle East (Table S1). The protein set for the reference *Candida albicans* strain SC5314 was also obtained from GenBank [41]. Construction of the *Candida albicans* pan-genome dataset was performed as detailed above, with potentially dubious gene model predictions for each genome checked against a dataset of 152 known dubious gene models from *Candida albicans* SC5314 obtained from the *Candida* Genome Database [48]. The completeness of each strain’s gene model dataset was assessed using 1711 *Saccharomyces cerevisiae* BUSCOs from the Saccharomycetales dataset; on average ~1642 BUSCOs (~96 %) were retrieved as complete gene models in each strain (Table S1). In total, 203786 gene models and their corresponding unique genomic locations were predicted for 34 *Candida albicans* genomes (Table S1).

### *Cryptococcus neoformans* var. *grubii*

Genomic data for 25 *Cryptococcus neoformans* var. *grubii* strains were obtained from the NCBI’s GenBank facility, encompassing both clinical and wild-type strains sampled from North America and Southern African regions (Table S1). The protein set for the reference *Cryptococcus neoformans* var. *grubii* strain H99 was also obtained from GenBank [42]. Construction of the *Cryptococcus neoformans* var. *grubii* pan-genome dataset was performed as detailed above, with the exception that a check for known dubious gene models was not carried out as no such data were available for *Cryptococcus neoformans* var. *grubii*. The completeness of each strain’s gene model dataset was assessed using the 1335 BUSCOs from the Basidiomycota dataset; on average ~987 BUSCOs (~74 %) were retrieved as complete gene models in each strain (Table S1). In total, 170241 gene models and their corresponding genomic locations were predicted for 25 *Cryptococcus neoformans* var. *grubii* genomes (Table S1).

### Aspergillus fumigatus

Genomic data for 12 *Aspergillus fumigatus* strains were obtained from the NCBI’s GenBank facility, including both clinical and wild-type strains isolated from the Northern and Southern hemispheres, and the International Space Station (Table S1). The protein set for the reference *Aspergillus fumigatus* strain AF293 was also obtained from GenBank [43]. Construction of the *Aspergillus fumigatus* pan-genome dataset was performed as detailed above, with the exception that a check for known dubious gene models was not carried out as no such data was available for *Aspergillus fumigatus*. The completeness of each strain’s gene model dataset was assessed using 4046 *Aspergillus nidulans* BUSCOs from the Eurotiomycetes dataset; on average ~3410 BUSCOs (~84 %) were retrieved as complete gene models in each strain (Table S1). In total, 116230 putative proteins and their corresponding unique genomic locations were predicted for 12 *Aspergillus fumigatus* genomes (Table S1).

### Pan-genome analysis of fungal species

Analysis of the pan-genomes of the four fungal species in our study was performed using the Perl software PanOCT [38]. PanOCT is a graph-based method that uses both blast score ratio [52] and CGN [53] approaches to establish clusters of syntenically conserved orthologues across multiple genomes for species pan-genome analysis (Fig. S1). The use of genomic context in addition to sequence similarity in PanOCT allowed us to distinguish between multiple homologous sequences within any genome analysed (i.e. paralogues) [38]. We used CGN (window size=5, the default value) as our criterion for defining conserved gene evolution between strains of fungal species. In the sections below, we refer to gene models containing an orthologue from all strains present in a species dataset as core gene models (and thus part of the core genome) and those missing an orthologue from one or more strains as accessory clusters (and thus part of the accessory genome). After removing invalid or low-quality blastp hits in each species dataset (Table S1), the initial core and accessory genomes for each species dataset were constructed using PanOCT with the default parameters.

To assess the influence of duplication and microsynteny loss on fungal pan-genomes, we processed the results of the PanOCT analysis using a multi-step Python/R post-processing pipeline. This first step of this pipeline was an iterative search for independent syntenic clusters with the potential to be merged based on reciprocal sequence similarity. Starting with accessory clusters of size *n* – 1 (where *n* is the number of strains in a dataset), parallelized all-vs-all blastp searches of all remaining gene models from accessory clusters (e=10^−4^) were performed, and this output was parsed to identify instances where two accessory clusters with no overlapping strain representation could be merged into one cluster based on the following criteria. (i) Each member gene model in a ‘query’ cluster of size *m* had a reciprocal blastp strain top hit with a sufficient number of member gene models in a ‘subject’ cluster of size *n – m* or smaller. (ii) The size of the resulting ‘merged’ cluster was ≤*n*.

This approach attempted to account for loss-of-synteny events such as rearrangements or other artefacts arising from different genome sequencing and assembly methods. Merged accessory clusters that now had an orthologous gene model from each strain in a dataset (i.e. whose size=*n*) were recategorized as core clusters, although for this study such recategorizations were a rare occurrence.

The second step of our post-processing pipeline assessed the influence of gene duplication on fungal pan-genome evolution by analysing the proportion of accessory gene models that were potentially paralogous to the core genome. Gene models from accessory clusters were assessed for sequence similarity to core gene models from the initial all-vs-all blastp search used as input for PanOCT. If accessory gene models were sufficiently similar to every gene model from a given core cluster (*E* value cut-off of 1e^−4^), then that accessory cluster was classified as being a paralogous cluster or a cluster of duplicated core gene models. This approach attempted to account for duplication events followed by subsequent gene loss, rearrangement in strains or strain-/lineage-specific expansions of gene families. Using a sequence-based approach of pan-genome analysis, as opposed to genome alignment or other methods, also facilitated the downstream application of systematic functional analysis of species pan-genomes; e.g. gene ontology (GO)-slim enrichment, which is detailed below. We visualized the distribution of syntenic orthologues within fungal accessory genomes using the UpSet technique, an alternative to Venn or Euler diagrams, which visualizes intersections of sets and their occurrences using a matrix representation [54]. This technique, implemented in the R package UpSetR, allowed us to see the number of shared syntenic orthologues (intersections) across different strains (sets) within a species dataset [55, 56]. Singleton gene models from each reference strain genome were functionally characterized by searching against their corresponding reference protein set using blastp (*e*=10^−4^).

### Phylogenomic reconstruction of intraspecific phylogenies

Phylogenomic reconstruction of intraspecific lineages was carried out for all four fungal species using a supermatrix approach. For each fungal pan-genome dataset, all core orthologue clusters whose smallest gene model was at least 90 % the length of the longest gene model were retrieved from the dataset. Each cluster was aligned in muscle with the default parameters, and for each cluster alignment phylogenetically informative character sites were extracted using paup* [57, 58]. Sampled alignments retaining character data were concatenated into a superalignment using FASConCAT [59].

In total, (i) 4311 *Saccharomyces cerevisiae* core clusters (431 100 gene models) passed the minimum sequence length criterion and retained alignment data after sampling, and were concatenated into a 100 genome superalignment containing 54 860 aa sites.

(ii) 4327 *Candida albicans* core clusters (68 904 gene models) retained alignment data after sampling, and were concatenated into a 34 genome superalignment containing 31 999 aa sites.

(iii) 4512 *Cryptococcus neoformans* var. *grubii* core clusters (112 800 gene models) retained alignment data after sampling, and were concatenated into a 25 genome superalignment containing 47 811 aa sites.

(iv) 5 724 *Aspergillus fumigatus* core clusters (68 904 gene models) retained alignment data after sampling for phylogenetically informative residues, and were concatenated into a 12 genome superalignment containing 20 760 aa sites.

**Fig. 2.**
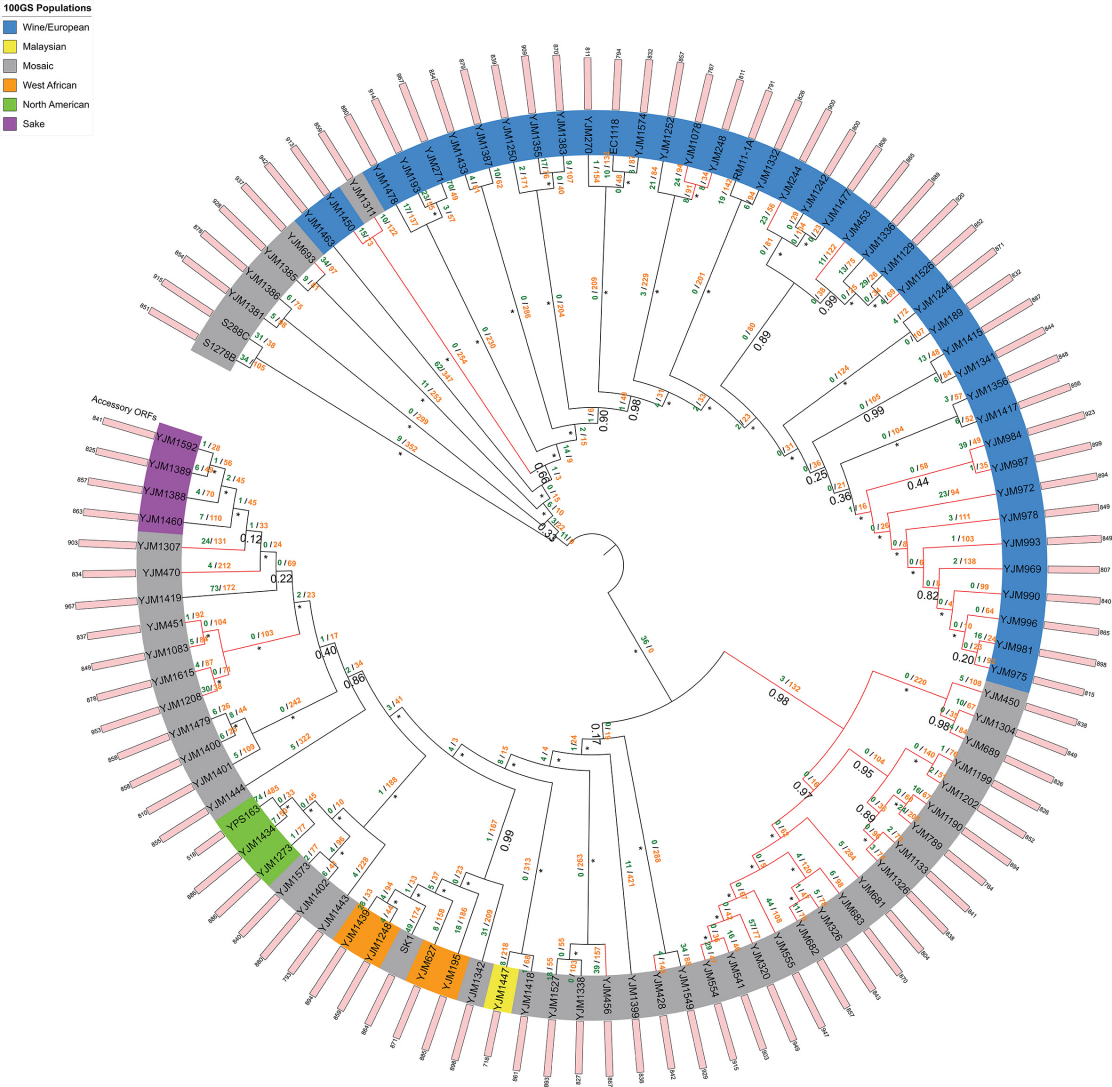
Approximate maximum-likelihood supermatrix phylogeny of the *Saccharomyces cerevisiae* pan-genome dataset based on 4311 core orthologue clusters. *Saccharomyces cerevisiae* populations are as assigned by Strope *et al*., clinical strains are indicated by red branches. Numbers below branches refer to Shimodaira–Hasegawa local supports, maximum supports are indicated by asterisks. Dollo parsimony analysis of gene model gain/loss events is annotated above branches in green and orange, respectively.

**Fig. 3.**
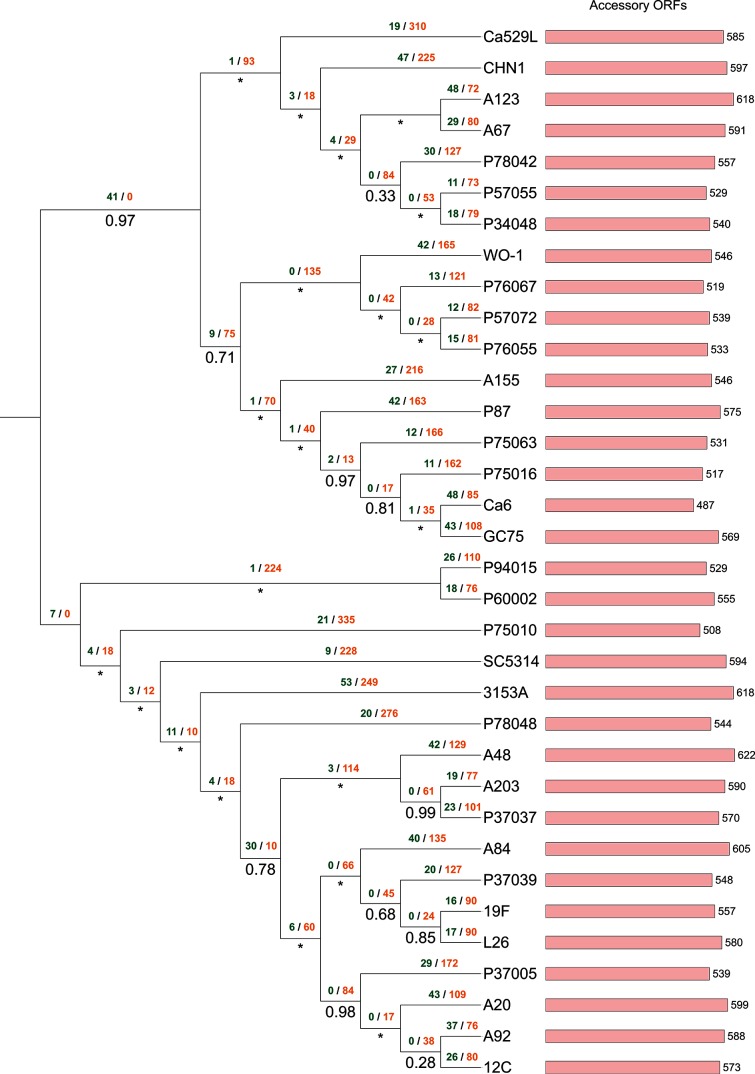
Approximate maximum-likelihood supermatrix phylogeny of the *Candida albicans* pan-genome dataset based on 4327 core orthologue clusters. Numbers below branches refer to Shimodaira–Hasegawa local supports, maximum supports are indicated by asterisks. Dollo parsimony analysis of gene model gain/loss events is annotated above branches in green and orange, respectively.

**Fig. 4.**
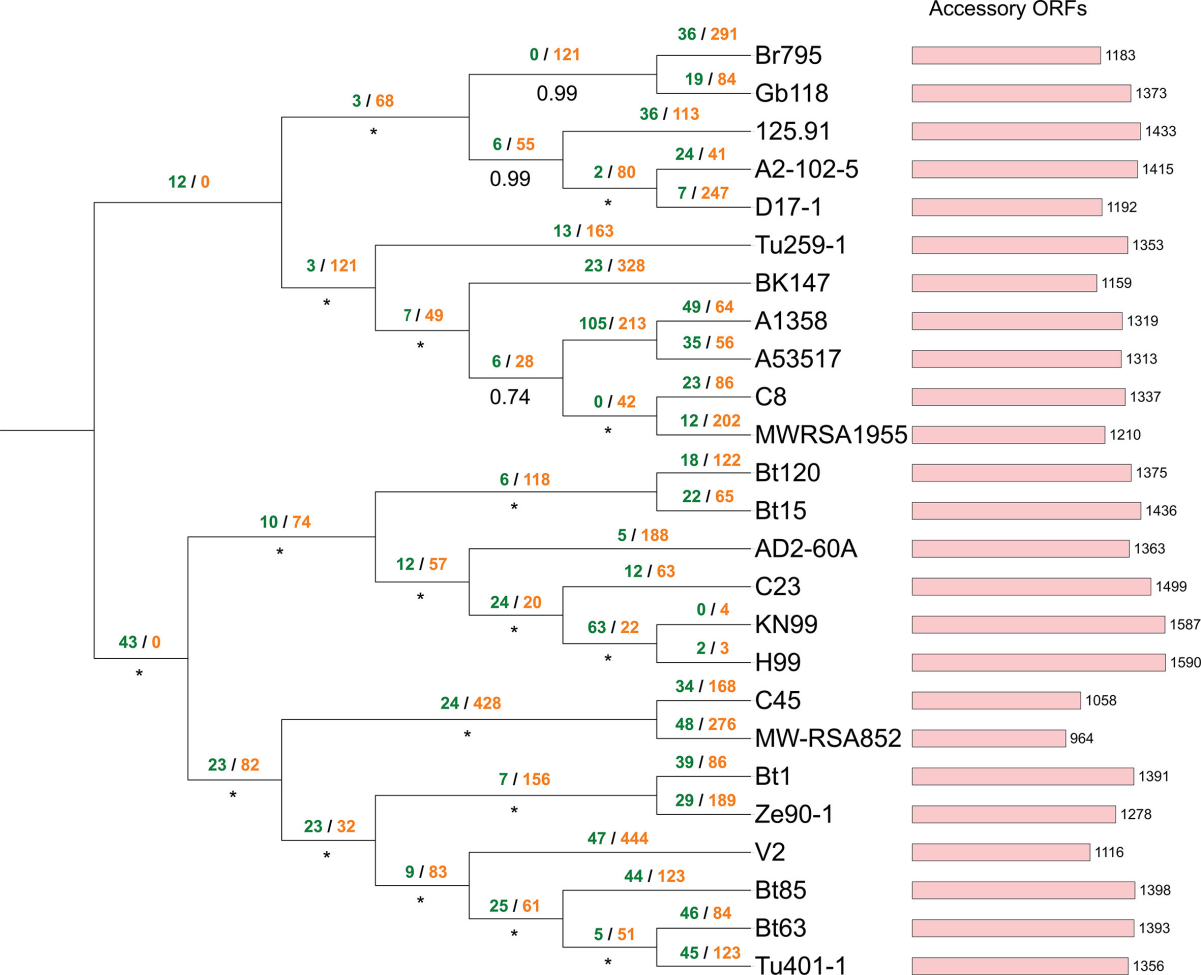
Approximate maximum-likelihood supermatrix phylogeny of the *Cryptococcus neoformans* var. *grubii* pan-genome dataset based on 4512 core orthologue clusters. Numbers below branches refer to Shimodaira–Hasegawa local supports, maximum supports are indicated by asterisks. Dollo parsimony analysis of gene model gain/loss events is annotated above branches in green and orange, respectively.

**Fig. 5.**
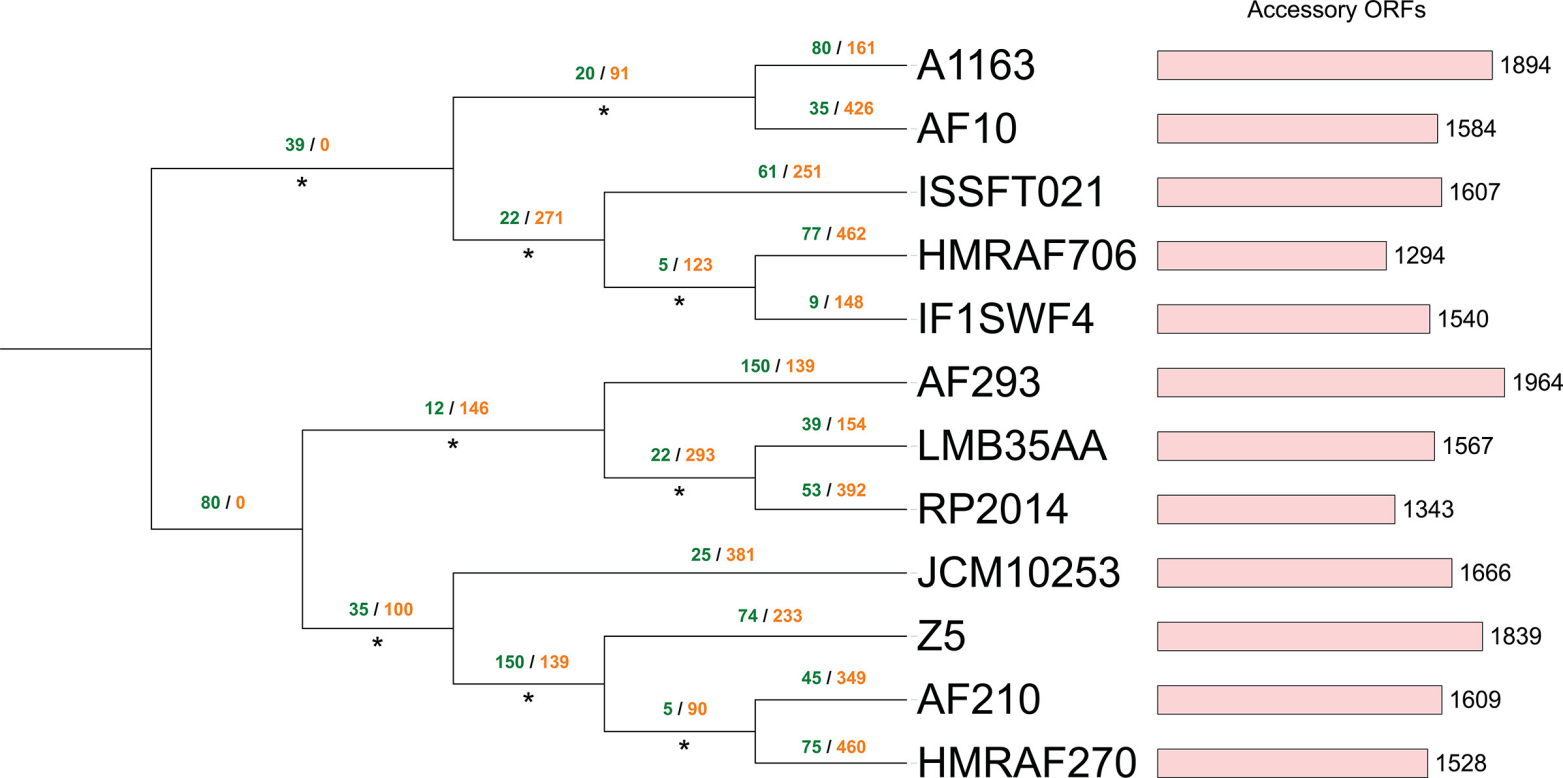
Approximate maximum-likelihood supermatrix phylogeny of the *Aspergillus fumigatus* pan-genome dataset based on 5724 core orthologue clusters. Numbers below branches refer to Shimodaira–Hasegawa local supports, maximum supports are indicated by asterisks. Dollo parsimony analysis of gene model gain/loss events is annotated above branches in green and orange, respectively.

Approximate maximum-likelihood phylogenomic reconstruction was performed for each superalignment using FastTree with the default JTT+CAT evolutionary model and Shimodaira–Hasegawa local supports [60]. All phylogenomic trees were rooted at the midpoint and annotated using the iTOL website [61] (Figs 2–5). A binary matrix was generated for the presence/absence of all orthologue clusters across all strains within each species accessory genome. Each species matrix was mapped onto the corresponding intraspecific supermatrix phylogeny and Dollo parsimony analysis was performed on each matrix using Count (Figs 2–5) [62, 63]. Orthologue gain and loss events were manually annotated onto each intraspecific phylogeny.

### Functional annotation and GO enrichment analysis of fungal species pan-genomes

Pfam, InterPro and GO annotation for all four fungal datasets was carried out using InterProScan [64–67]. The total numbers of proteins with at least one annotation per database from the original putative protein sets per species are given in Table 1. Enrichment analysis of GO terms was carried out for the core and accessory complements of each species’ pan-genome by mapping all GO terms per species to their species GO-slim counterparts (or to the general GO-slim term basket for *Cryptococcus neoformans* var. *grubii*) and performing a Fischer’s exact test analysis with parent term propagation and false discovery rate correction (*P*<0.05) for all complements using the Python package GOAtools (Table S2) [67–69]. False discovery rate correction was applied for all Fischer’s exact tests in GOAtools using a *P* value distribution generated from 500 resampled *P* values.

**Table 1.** Number of gene models in our four fungal pan-genome datasets with at least one annotation term per annotation type Percentage of annotated gene models relative to pan-genome datasets shown in parentheses.

Species	Pfam	InterPro	GO
*Saccharomyces cerevisiae*	468 511 (81 %)	455 582 (79 %)	312 161 (54 %)
*Candida albicans*	161 235 (79 %)	155 271 (76 %)	105 694 (52 %)
*Cryptococcus neoformans*	111 305 (65 %)	106 655 (63 %)	72 243 (42 %)
*Aspergillus fumigatus*	83 239 (71 %)	79 231 (68 %)	54 457 (46 %)

### Putative ancestral history of fungal core and accessory genomes

The putative evolutionary history of fungal core and accessory genomes was analysed by querying all gene models per species against a >5 million protein dataset sampled from 1109 bacterial and 488 archaeal genomes obtained from UniProt, using blastp with an *E* value cut-off of 10^−20^ [70]. Gene models were filtered by their ancestral history into three classifications using the following criteria. (i) Gene models whose hits were exclusively from bacterial or archaeal sequences were classified as ‘bacterial’ or ‘archaeal’ in origin, respectively. (ii) Gene models whose hits contained both bacterial and archaeal sequences were classified as ‘undefined prokaryote’ in origin. (iii) Gene models that did not hit any protein sequence in the dataset were classified as ‘eukaryotic’ in origin (Table S3). Pearson’s χ^2^ tests were carried out to determine the significance of prokaryote and eukaryote origin frequencies within the complements of each species pan-genome [68] (Table S3).

### Extent of HGT in fungal accessory genomes

The extent of HGT in each fungal accessory genome was assessed by randomly selecting representative gene models from each accessory cluster and searching these using blastp with an *E* value cut-off of 1e^−20^ against a dataset representative of fully sequenced prokaryotic and eukaryotic species. This dataset was composed of over 8 million protein sequences from 1698 genomes sampled from all three domains of life that had been used in previous interdomain HGT analysis [71], as well as all predicted gene models per species dataset. Putative interdomain HGT events were identified by locating gene models whose first top hit outside either the sequence’s source species or genus was prokaryotic in origin. Putative HGT events identified by either filter are given per species in Table S3. Putative intrakingdom fungal HGT events were identified by filtering the same blastp output for gene models whose first top hit outside the sequence’s source species was fungal in origin but not from the same genus (Table S3).

### Chromosomal location of core and accessory gene models in species reference genomes

Pearson’s χ^2^ tests were carried out for the global frequencies of core and accessory gene models along the subterminal regions of chromosomes, which we defined as approximately the first and last 10 % of each chromosome, in each reference genome (Table S4). Pearson’s χ^2^ tests were also carried out for the frequencies of core and accessory gene models per chromosome for each reference genome (Table S4) [68]. The chromosomal locations of core and accessory gene models along each reference genome were visualized using the Ruby software PhenoGram [72].

### Distribution of knockout viability phenotypes in *Saccharomyces cerevisiae* S288C

All available knockout phenotype data for *Saccharomyces cerevisiae* S288C were obtained from the SGD [73]. A reciprocal blastp search was carried out between all 5815 *Saccharomyces cerevisiae* S288C gene models from our *Saccharomyces cerevisiae* pan-genome dataset and the reference protein set for *Saccharomyces cerevisiae* S288C with an *E* value cut-off of 10^−20^ to match predicted proteins to orthologues from the reference protein set. Knockout phenotype viability data, if available, was then inferred for each of our *Saccharomyces cerevisiae* S288C gene models that had a reciprocal reference orthologue. Pearson’s χ^2^ tests were carried out for the frequencies of knockout phenotype viability in both the core and accessory genomes of *Saccharomyces cerevisiae* S288C (Table S5).

### Distribution of ‘dispensable pathway’ (DP) genes in the *Saccharomyces cerevisiae* pan-genome

Data for 14 DP gene clusters containing 41 genes found in *Saccharomyces cerevisiae* was taken from a previously published analysis of biotin reacquisition in yeast species [74]. A total of 38 DP genes were extracted from the *Saccharomyces cerevisiae* S288C reference protein set, encompassing 13 of the 14 DP clusters. A reciprocal blastp search was performed between these genes and all 5815 *Saccharomyces cerevisiae* S288C gene models from the *Saccharomyces cerevisiae* pan-genome dataset with an *E* value cut-off of 10^−20^ to identify DP genes in our predicted gene model set. All 38 DP genes had a unique reciprocal match with a predicted gene model in *Saccharomyces cerevisiae* S288C. A binary matrix was generated for the presence/absence of syntenic orthologues of DP genes from *Saccharomyces cerevisiae* S288C in the *Saccharomyces cerevisiae* pan-genome dataset (Table S5).

### Distribution of biosynthetic gene clusters (BGCs) in the *Aspergillus fumigatus* pan-genome

Data for 33 known BGCs encompassing 307 genes in *Aspergillus fumigatus* Af293 were obtained from a previous analysis of secondary metabolism in *Aspergillus fumigatus* [75]. *Aspergillus fumigatus* Af293 gene models from the *Aspergillus fumigatus* pan-genome dataset were matched to their homologues from the reference gene data set using a reciprocal blastp search with an *E* value cut-off of 10^−20^. A binary matrix was constructed for the presence/absence of syntenic orthologues of the 307 putative BGC genes from *Aspergillus fumigatus* Af293 within the *Aspergillus fumigatus* pan-genome dataset (Table S5).

## Results

### Analysis of the *Saccharomyces cerevisiae* pan-genome

**Fig. 6.**
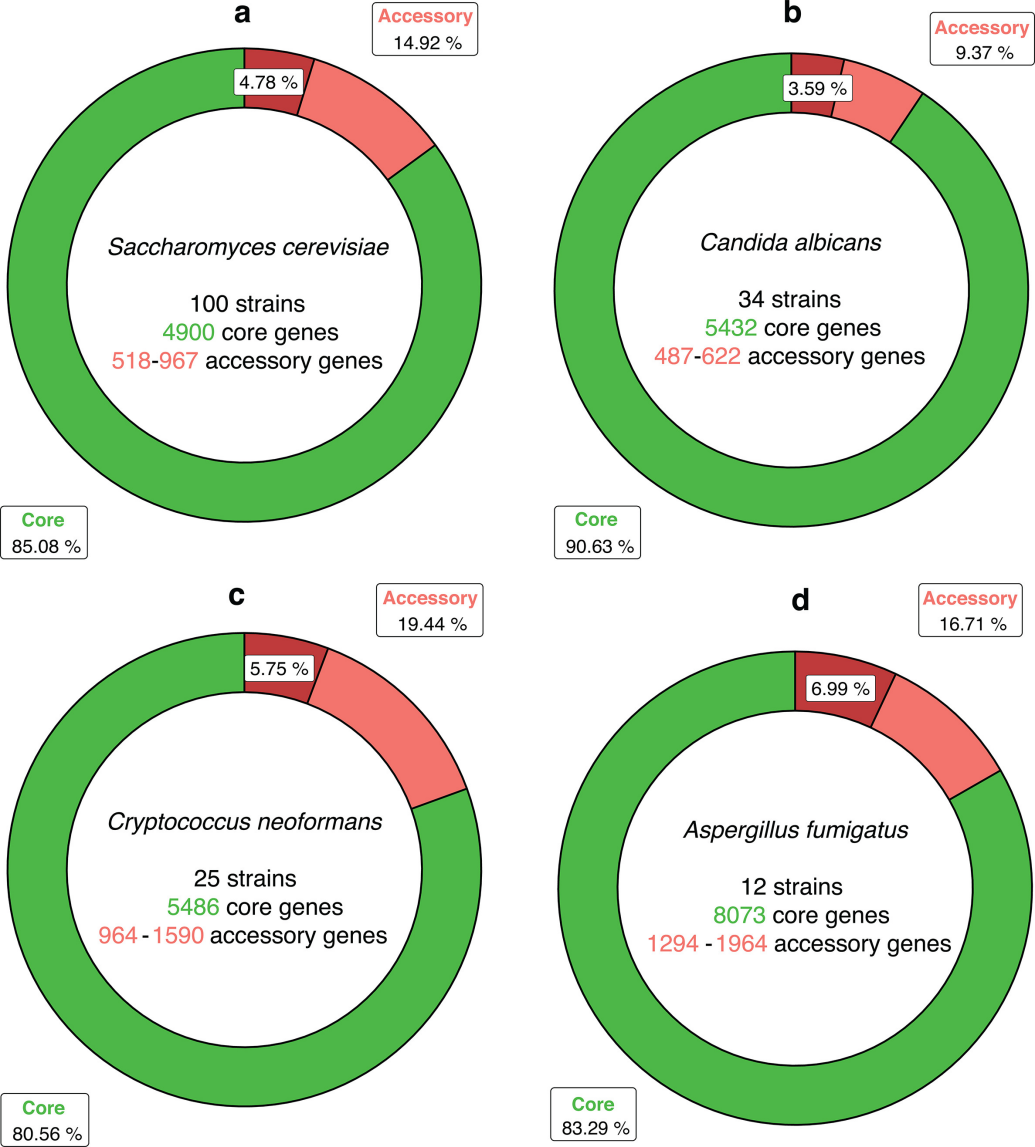
Pan-genomes of four fungal species. (a) *Saccharomyces cerevisiae*, (b) *Candida albicans*, (c) *Cryptococcus neoformans* var. *grubii*, (d) *Aspergillus fumigatus*. The ring charts represent the total number of gene models in pan-genome complements expressed as a proportion of total pan-genome size. Sections in dark-red represent duplicated core gene models in the accessory genome.

Overall, 575 940 gene models were predicted across all 100 *Saccharomyces cerevisiae* strains with a mean of 5759 gene models predicted per strain (Tables 2 and S1). These 575 940 gene models were distributed across 7750 unique syntenic orthologue clusters (Table 2). The core *Saccharomyces cerevisiae* genome contained 4900 gene models, which were conserved across 100 *Saccharomyces cerevisiae* strains (490 000 gene models in total, 85 % of the total species pan-genome). For individual strain genomes, this corresponded to between 83 and 90 % of their total predicted gene model content (Fig. 6a, Table S1). The remaining 85 940 predicted gene models were accessory gene models, distributed across 2850 clusters, with strain accessory genome sizes ranging from 518 to 967 gene models per *Saccharomyces cerevisiae* strain (mean size =~859 gene models). Further analysis of the *Saccharomyces cerevisiae* species accessory genome identified that ~32 % of accessory gene models (776 clusters, 4.77 % of the total species pan-genome) were duplicates of core gene models conserved across one or more strains. This corresponded to a mean of 275 gene models per *Saccharomyces cerevisiae* strain, and 27 511 gene models in total (Tables 2 and S1). Overall, 455 syntenic clusters (encompassing 45 045 accessory gene models) were missing a syntenic orthologue in only one other strain and 1416 accessory gene models were singletons. Analysis of the distribution of orthologues within the *Saccharomyces cerevisiae* accessory genome using the R package UpSetR showed that the most frequent sets are singleton gene models or syntenic clusters missing a syntenic orthologue in one strain, with YPS163 having the most singleton genes (74 in total) (Fig. S3). Other strains (e.g. YJM1477) lacked singleton gene models altogether (Fig. 2). There were 13 756 gene models (from 1935 syntenic clusters) that did not have a syntenic orthologue in *Saccharomyces cerevisiae* S288C. Of these non-reference gene models, 1385 were singleton gene models found only in one strain. The widest-distributed non-reference gene model was present in 93 strains and there was no accessory gene model solely missing from *Saccharomyces cerevisiae* S288C. YPS163 had the smallest accessory genome of the 100 yeast strains (518 gene models) and YJM271 had the largest (967 gene models) (Fig. 2).

Phylogenomic reconstruction of all 100 *Saccharomyces cerevisiae* strains resolved two major groups; a clade containing strains and mosaics derived from Malaysian, West African, North American and sake populations, and a clade containing strains and mosaics derived from wine/European populations (Fig. 2). Each of the non-mosaic populations as assigned by Strope *et al.* [50] present in the dataset (except the singleton Malaysian strain YJM1447) resolved to a monophyletic geographical group [50]; the placement of the mosaic laboratory strain SK-1 in a West African clade is consistent with its West African origin [76], and the clinical mosaic strain YJM1311 is of predominantly wine/European ancestry; hence, its placement at the base of the wine/European clade [50] (Fig. 2). Many of the remaining mosaic strains branched close to non-mosaic clades that shared their dominant population fraction as determined by Strope *et al.* [50]; for example, many of the clinical mosaic strains placed adjacent to the sake clade had predominantly sake population ancestry [50] (Fig. 2). Three strains (YJM248, YJM1252, YJM1078) identified by Strope *et al*. [50] as having a higher relative proportion of introgressed genes than other *Saccharomyces cerevisiae* strains (potentially arising from recent hybridization with *Saccharomyces paradoxus*) formed a monophyletic branch within the previously described wine/European clade [50].

### Analysis of the *Candida albicans* pan-genome

A total of 203 786 gene models were predicted across all 34 *Candida albicans* strain genomes, with a mean of 5993 gene models predicted per strain, distributed across 7325 unique syntenic orthologue clusters (Tables 2 and S1). The core *Candida albicans* genome contained 5432 gene models that were conserved across 34 *Candida albicans* strains (184  688 in total, 90 % of the total species pan-genome). This corresponded to between 89 and 91 % of the total predicted gene models for each strain genome (Fig. 6b, Table S1). The remaining 19 098 predicted gene models were accessory gene models, distributed across 1893 clusters, with strain accessory genome sizes ranging from 487 to 622 gene models per *Candida albicans* strain (mean size =~561 gene models) (Tables 2 and S1). Further analysis of the *Candida albicans* species accessory genome identified that ~38 % of accessory gene models (1013 clusters, ~3.59 % of the total species pan-genome) were duplicates of core gene models conserved across one or more strains. This corresponded to a mean of 215 gene models per *Candida albicans* strain, and 7312 gene models in total (Tables 2 and S1). Of the 19 098 *Candida albicans* accessory gene models identified, 3624 accessory gene models (from 268 syntenic clusters) were missing a syntenic orthologue in only one other strain, while 928 gene models were singletons. UpSet analysis of the distribution of orthologues within the *Candida albicans* accessory genome showed that 1056 gene models (32 syntenic clusters) from 33 *Candida albicans* strains were missing an orthologue in *Candida albicans* WO-1 and *Candida albicans* 3153A had 53 putative gene models with no orthologue in any other strain (Fig. S4). SC5314 had the smallest number of singleton gene models (nine in total). *Candida albicans* A48 had the largest accessory genome (622 gene models) and *Candida albicans* Ca6 had the smallest (487 gene models) (Fig. 3). Phylogenomic reconstruction of all 34 *Candida albicans* strains resolved two main groups when rooted at the midpoint; one containing the exemplar *MTL*-homozygous strain WO-1 and a ladderized group containing the reference strain SC5314 (Fig. 3).

**Table 2.** Pan-genomes of four fungal species: *Saccharomyces cerevisiae, Candida albicans*, *Cryptococcus neoformans* var. *grubii* and *Aspergillus fumigatus* Duplicated core gene models (GMs) and clusters in the accessory genomes are given in parentheses.

Species	Strains	Core genome		Accessory genome		Pan-genome	
		GMs	Clusters	GMs	Clusters	GMs	Clusters
*Saccharomyces cerevisiae*	100	490 000	4900	85 940 (27 511)	2850 (776)	575 940	7750
*Candida albicans*	34	184 688	5432	19 098 (7312)	1893 (1013)	203786	7325
*Cryptococcus neoformans*	25	137 150	5486	33 091 (9974)	2698 (776)	170241	8193
*Aspergillus fumigatus*	12	96 876	8073	19 435 (8127)	3002 (1170)	116 311	11 075

### Analysis of the *Cryptococcus neoformans* var. *grubii* pan-genome

A total of 170 241 gene models were predicted across all 25 *Cryptococcus neoformans* var. *grubii* strain genomes, with a mean of 6809 gene models predicted per strain, distributed across 8193 unique syntenic orthologue clusters (Tables 2 and S1). The core *Cryptococcus neoformans* var. *grubii* genome contained 5486 gene models that were conserved across 25 *Cryptococcus neoformans* var. *grubii* strains (137 150 in total, 80 % of the total species pan-genome). This corresponded to between 76 and 85 % of the total predicted gene models for each strain genome (Fig. 6c, Table S1). The remaining 33 091 predicted gene models were accessory gene models distributed across 2698 clusters, with strain accessory genome sizes ranging from 964 to 1654 gene models per *Cryptococcus neoformans* var. *grubii* strain (mean size =~1334 gene models) (Table S1). Detailed analysis of the *Cryptococcus neoformans* var. *grubii* species accessory genome identified that ~29 % of accessory gene models (776 clusters, ~5.8 % of the total species pan-genome) were duplicates of core gene models conserved across one or more strains. This corresponded to a mean of ~391 gene models per *Cryptococcus neoformans* var. *grubii* strain, and 9794 gene models in total (Tables 2 and S1). Overall 674 *Cryptococcus neoformans* var. *grubii* clusters (encompassing 16 032 accessory gene models) were missing a syntenic orthologue in only one other strain and 668 accessory gene models were singletons. UpSet analysis of the distribution of orthologues within the *Cryptococcus neoformans* var. *grubii* accessory genome showed that 3600 gene models (150 syntenic clusters) from 24 *Cryptococcus neoformans* var. *grubii* strains were missing an orthologue in *Cryptococcus neoformans* var. *grubii* MWRSA852, whereas the *Cryptococcus neoformans* var. *grubii* A1358 genome had 49 putative gene models with no orthologue in any other strain (Fig. S5). KN99 had no singleton gene models, but it should be noted that that strain is an isogenic derivative of the reference H99 strain. *Cryptococcus neoformans* var. *grubii* H99 itself had the largest accessory genome (1590 gene models) and *Cryptococcus neoformans* var. *grubii* MW-RSA852 had the smallest (964 gene models) (Fig. 4). The most frequent sets found in the accessory genome include both singleton genes and clusters missing orthologues from one or two strains. Phylogenomic reconstruction of all 25 strains using a 47 811-site amino acid supermatrix derived from the core *Cryptococcus neoformans* var. *grubii* genome resolved two monophyletic groups when rooted at the midpoint (Fig. 4).

### Analysis of the *Aspergillus fumigatus* pan-genome

A total of 116 311 gene models were predicted across all 12 *Aspergillus fumigatus* strain genomes, distributed across 11 075 unique syntenic orthologue clusters, with a mean of 9692 gene models predicted per strain. The core *Aspergillus fumigatus* genome contained 8073 core gene models that were conserved across 12 *Aspergillus fumigatus* strains (96 876 in total, 83 % of the total species pan-genome). This corresponded to between 80 and 86 % of the total predicted gene models for each strain genome (Fig. 6d, Table S1). The remaining 19 435 predicted gene models were accessory gene models distributed across 3002 clusters, with strain accessory genome sizes ranging from 1294 to 1964 gene models per *Aspergillus fumigatus* strain (mean size =~1619 gene models) (Table S1). Detailed analysis of the *Aspergillus fumigatus* species accessory genome identified that ~41 % of accessory gene models (1170 clusters, ~6.9 % of the total species pan-genome) were duplicates of core gene models that were conserved across one or more strains. This corresponded to a mean of 677 gene models per *Aspergillus fumigatus* strain, and 8127 gene models in total. Overall, 7953 gene models (from 958 syntenic clusters) were missing a syntenic orthologue in only one other strain, whereas 723 gene models were singletons.

UpSet analysis of the orthologue distribution in the *Aspergillus fumigatus* accessory genome found that 2167 gene models (197 syntenic clusters) from 11 *Aspergillus fumigatus* strains were missing an orthologue in *Aspergillus fumigatus* IFISWF4 and the reference *Aspergillus fumigatus* Af293 genome has 150 putative gene models with no orthologue in any other strain (Fig. S6). The latter may be due to a lower degree of strain sampling within the *Aspergillus fumigatus* dataset or the reference genome having a higher-quality assembly than other strains of *Aspergillus fumigatus*. The Z5 strain has the smallest number of singleton gene models (nine in total). *Aspergillus fumigatus* Af293 has the largest accessory genome (1964 gene models) and *Aspergillus fumigatus* HMRAF706 has the smallest (1294 gene models) (Fig. 5). Phylogenomic reconstruction of all 12 strains using a 20 760-site amino acid supermatrix derived from the core *Aspergillus fumigatus* genome resolved two monophyletic groups when rooted at the midpoint, one containing both International Space Station strains and *Aspergillus fumigatus* Af10, and one containing all three environmental strains as well as *Aspergillus fumigatus* Af293 and Af210 (Fig. 5). The placement of the two International Space Station strains as well as the aforementioned individual clinical strains is in relative agreement with the most extensive intraspecific *Aspergillus fumigatus* phylogeny published [77].

### GO enrichment in fungal core and accessory genomes

Analysis of the distribution of GO terms in fungal core genomes shows that many housekeeping biological processes, such as translation, nucleic acid metabolism and oligopeptide metabolism, are significantly over-represented in each species (*P*<0.05) (Table S2). Furthermore, molecular function terms for enzymatic and nucleic acid binding activity are also significantly over-represented (Table S2). In fungal accessory genomes, terms relating to transport and localization of proteins, carbohydrate metabolism, as well as protein modification and carboxyl acid metabolism, are significantly over-represented in many species (Table S2). Terms relating to housekeeping processes are significantly under-represented in fungal accessory genomes compared to core genomes. There are no common or synonymous cellular component or molecular function terms that are significantly under-represented across all four fungal accessory genomes in our analysis. However, terms relating to the functions of intracellular membrane-bound organelles are significantly over-represented in the accessory genomes of both *Cryptococcus neoformans* var. *grubii* and *Aspergillus fumigatus* (Table S2).

Many broad and granular housekeeping terms relating to nucleic acid and protein biological processes are significantly over-represented within the core genome of *Saccharomyces cerevisiae* (Table S2). In addition to transport processes, genes potentially involved in vitamin metabolism and protein dephosphorylation are significantly over-represented within the core genome of *Saccharomyces cerevisiae*. Similar terms are also significantly over-represented within the core genome of *Candida albicans* (Table S2). The *Cryptococcus neoformans* var. *grubii* core genome is significantly over-represented in some unique terms involved in regulation of homeostasis and biological quality, functional pathways such as the unfolded protein response (UPR) pathway, as well as signal transduction (Table S2). There are fewer terms that are significantly over-represented within the *Cryptococcus neoformans* var. *grubii* accessory genome than in the other fungal accessory genomes in this study. Those terms that are significantly over-represented in the *Cryptococcus neoformans* var. *grubii* accessory genome are also found elsewhere, e.g. transport. The core *Aspergillus fumigatus* genome is significantly over-represented in terms related to small molecule biosynthesis and other biosynthetic processes (Table S2). Within the *Aspergillus fumigatus* core genome, terms relating to vesicle-mediated transport and carboxylic acid metabolism are significantly over-represented, these terms are also significantly over-represented in the *Saccharomyces cerevisiae* core genome.

### Ancestral origin of fungal core and accessory genomes

The ancestral origin of fungal core and accessory genomes was inferred via blastp searches (1*e*^−20^) of fungal gene models against >5 million prokaryotic sequences from >1500 bacterial and archaeal genomes. Gene models that had hits with prokaryotic sequences exclusively were classified as having originated within the prokaryotes (broken down further by prokaryotic kingdom in Table S3), and gene models that lacked a blastp hit against the prokaryotic database were classified as having originated within the eukaryotes. Using these criteria, for each fungal pan-genome dataset between 69 and 77 % of all gene models were inferred as eukaryotic in origin. Similar proportions of gene models inferred as having originated within eukaryotes were also observed in fungal core genomes. Higher proportions of gene models with a putative origin within eukaryotes was observed in fungal accessory genomes (74–81 % of all accessory gene models in each species). Statistical analysis of the ancestral history of each fungal species pan-genome found that each fungal accessory genome was significantly enriched for genes of eukaryotic origin and each fungal core genome was significantly enriched for genes of prokaryotic origin (*P*<0.05) (Table S3).

### Interdomain and intrakingdom HGT into fungal accessory genomes

Systematic screening for interdomain HGT events in each fungal accessory genome revealed small numbers of putative HGT events from prokaryote sources per species, ranging from a single event in the *Candida albicans* accessory genome to 11 events in the *Aspergillus fumigatus* accessory genome (Table S3). The distribution of these putative HGT genes in fungal accessory genomes varies from strain-unique singleton genes (particularly in *Saccharomyces cerevisiae*) to more widely distributed genes (as seen in *Cryptococcus neoformans* and *Aspergillus fumigatus*) (Table S3). The majority of potential prokaryote donors are soil-dwelling bacteria, such as *Clostridium pasteurianum* (a donor to the *Aspergillus fumigatus* accessory genome) and *Acinetobacter pittii* (a donor to the *Saccharomyces cerevisiae* accessory genome). We then applied a similar screen for recent HGT from other fungal species, which suggested up to 8 % of fungal accessory genomes may have arisen via intrakingdom HGT. The largest extent of such intradomain HGT appeared to have occurred into the accessory genomes of *Cryptococcus neoformans* and *Aspergillus fumigatus* (420 and 391 potential events, respectively) (Table S3). In each accessory genome, putative HGT-derived gene models appear to have been transferred mainly from closely related species or species that share similar niches. For example, *Aspergillus fumigatus* is a potential donor of three *Candida albicans* accessory gene models (Table S3). However, further comprehensive investigations are required to confidently confirm that these HGT events are bona fide.

### Chromosomal location of core and accessory genomes in fungal reference genomes

Between 17 and 21 % of all predicted gene models for each fungal reference strain lie in the subterminal regions of that strain’s genome. Approximately 15 % of all core gene models in both *Saccharomyces cerevisiae* S288C and *Cryptococcus neoformans* var. *grubii* H99 are found in their subterminal regions, whereas this proportion is higher in *Candida albicans* SC5314 and *Aspergillus fumigatus* Af293 (~21 and ~18 % of all core gene models, respectively). *Candida albicans* SC5314 has a lower proportion of accessory gene models (115 of 594 gene models, ~19 % of its total accessory genome) found in subterminal regions than the other three fungal species, where that proportion is ~28–33 % of their total accessory genomes. There is a statistically significant bias (*P*<0.05) towards accessory gene models in the subterminal regions of *Saccharomyces cerevisiae* S288c, *Cryptococcus neoformans* var. *grubii* H99 and *Aspergillus fumigatus* Af293, with a corresponding bias (*P*<0.05) towards core gene models in the non-subterminal regions of each genome (Table S4). In contrast, there is no significant pattern in the distribution of accessory gene models in *Candida albicans* SC5314, and instead its subterminal regions are significantly enriched for core gene models (*P*<0.05) (Table S4). Statistical analysis of core and accessory gene model enrichment per chromosome in each reference genome found that at least one chromosome was significantly enriched for core gene models and another chromosome was significantly enriched for accessory gene models per genome (*P*<0.05) (Table S4). The number of chromosomes per genome that were significantly biased towards either core or accessory gene models ranged from two in *Candida albicans* SC5314 (chromosomes 2 and 7) to six in *Saccharomyces cerevisiae* S288C (chromosomes I–III, VI, VIII and XIII) (Table S4). Visualizing chromosomal plots showed that clustering of accessory genes mostly occurred in subterminal regions of fungal genomes (Fig. S7a–d). There are some exceptions: some chromosomes in *Saccharomyces cerevisiae* S288c, *Cryptococcus neoformans* var. *grubii* H99 and *Aspergillus fumigatus* Af293 had at least one larger accessory gene cluster closer to the chromosomal midpoint (Fig. S7a, c–d). In contrast, there appeared to be no major clustering of accessory genes in any chromosome in *Candida albicans* SC5314 (Fig. S7b).

### Knockout viability of core and accessory genes in *Saccharomyces cerevisiae* S288C

A total of 5343 predicted *Saccharomyces cerevisiae* S288C gene models from the species pan-genome dataset, encompassing 4730 core gene models and 613 accessory gene models, were assigned their reference homologue’s corresponding knockout viability phenotype. The remaining 472 predicted gene models from *Saccharomyces cerevisiae* S288C did not have a knockout viability phenotype assigned to them, either due to the lack of a unique reciprocal blastp hit or a lack of viability data for the reference homologue (Table S5). Those *Saccharomyces cerevisiae* S288C gene models that had knockout phenotype data were predominantly knockout-viable;~79 % of annotated core gene models and ~88 % of annotated accessory gene models had a reciprocal reference homologue with a viable knockout phenotype (Table S5). There was no significant bias in the distribution of knockout viability within the core *Saccharomyces cerevisiae* S288C genome, i.e. the core genome was enriched for neither knockout-viable or knockout-inviable gene models (of those which had knockout phenotype data available) (Table S5). The *Saccharomyces cerevisiae* S288C accessory genome, however, was over-represented for knockout-viable gene models (*P*<0.05) (Table S5).

### DP gene clusters in the *Saccharomyces cerevisiae* pan-genome

All 38 reference DP genes had a unique reciprocal homologue within the set of predicted *Saccharomyces cerevisiae* S288C gene models taken from our pan-genome dataset (Table S5). One of the 13 reference DP clusters was syntenically conserved within all strains in the *Saccharomyces cerevisiae* pan-genome dataset; a three-member *GAL* cluster involved in galactose utilization. Some clusters are widely conserved within the dataset, but are missing a member gene in a small number of strains; these include a three-member *BIO* cluster that mediates biotin uptake, a *SNO1-SNZ1* vitamin B6 metabolism cluster and a large six-member *DAL-DCG* cluster that enables utilization of allantoin as a nitrogen source (Table S5). Other clusters had more patchy distribution within the species pan-genome, most notably a three-member *ARR* gene cluster that confers arsenic resistance was missing a member gene (*ARR3*) in 49 out of 100 strains (Table S5). Some clusters, such as a four-member *FIT*/*FRE* iron uptake cluster, are completely missing in a small number of strains (Table S5).

### BGCs in the *Aspergillus fumigatus* pan-genome

A total of 307 known biosynthetic genes from 33 BGCs in *Aspergillus fumigatus* Af293 had a unique reciprocal homologue within the set of predicted *Aspergillus fumigatus* Af293 gene models from the *Aspergillus fumigatus* pan-genome [75]. A total of 240 of the 307 known biosynthetic genes were core genes found in all 12 *Aspergillus fumigatus* strains, none of which were unique to *Aspergillus fumigatus* Af293 alone (Table S5). There were 14 *Aspergillus fumigatus* BGCs that were completely conserved (i.e. all genes within that cluster are core genes), which included known mycotoxin-producing BGCs such as fumagillin and gliotoxin clusters (Table S5). Other BGCs were found to have one or two genes missing, potentially due to synteny loss or pseudogenization. Some BGCs showed far more variable distribution within the *Aspergillus fumigatus* pan-genome; for example, a polyketide synthase (PKS) cluster was wholly conserved in four strains (Af293, Z5, HMRAF270 and JCM10253) and absent or translocated in the other eight, and a fusarielin-like cluster was completely absent from A1163 and only partially present in some strains but was wholly conserved in others (Table S5).

## Discussion

### Applying genomic context in eukaryotic pan-genome analysis

To investigate pan-genomic structure within four fungal species, we adapted a method previously used in bacterial pan-genome analysis and implemented in PanOCT (Pan**-**genome Ortholog Clustering Tool) [38]. Our rationale for using this method to construct species pan-genomes was that it allowed us to investigate intraspecific variability on a gene-to-gene level, as opposed to defining core and accessory genomes based on families of related gene models (e.g. a core gene family may be present in all strains of a species, but the number of genes belonging to that family will usually vary between strains). This allowed us to see which genes and biological functions were relatively conserved in their distribution, and which had varying expansion and distribution in fungal species. A similar approach was used in a previous analysis of genome variation in *Saccharomyces* species, but was limited to assessing syntenic conservation of reference homologues using immediately adjacent genes [34]. To ensure consistency between strain genomes in each of our datasets we constructed a custom gene model prediction pipeline that used three different predictive methods to generate a unique set of predicted gene models and their genomic locations (i.e. no isoforms) per strain genome (Fig. S2) [44–46]. As our definition of what constitutes a core or accessory gene model is quite stringent compared to other pan-genome analyses, we also developed a post-processing pipeline that attempted to account for loss of microsynteny between fungal strain genomes and to also examine the extent of duplication of core genome content within fungal accessory genomes.

### Pan-genomes of four model fungi

We chose to investigate the potential pan-genomic structure of four model fungal species: *Saccharomyces cerevisiae*, *Candida albicans*, *Cryptococcus neoformans* var. *grubii* and *Aspergillus fumigatus*. In addition to their impact on human lifestyle, each species chosen is a model organism for fungal evolutionary biology, genomics and comparative genomics. *Saccharomyces cerevisiae* was the first eukaryote to have its genome sequenced, and the other three species each had their genome sequenced during the initial wave of fungal genomics research in the early to mid-2000s [39–43, 78]. Our selection covers fungal species with different genomic characteristics; *Saccharomyces cerevisiae* has undergone ancestral whole-genome duplication and *Candida albicans* has an alternative genetic code [79, 80], whereas *Cryptococcus neoformans* and *Aspergillus fumigatus* are more intron-dense than either *Saccharomyces cerevisiae* or *Candida albicans* and extensive alternative splicing occurs in *Cryptococcus* species [81, 82]. Our selection also covers fungal species with different evolutionary histories. *Saccharomyces cerevisiae*, *Candida albicans* and *Aspergillus fumigatus* are members of the fungal phylum Ascomycota; the former two are closely related members of the subphylum Saccharomycotina, which includes many typical commensal and pathogenic yeasts that reproduce by budding, while *Aspergillus fumigatus* is a member of the large subphylum Pezizomycotina of filamentous fungi [78]. *Cryptococcus neoformans* var. *grubii* superficially resembles many yeast species and also replicates by budding, but is a member of the phylum Basidiomycota and is more closely related to multicellular fungi within the subphylum Agaricomycotina than other yeast species [78]. Genome assemblies available on GenBank for each species at the time of writing ranged from 12 for *Aspergillus fumigatus* to >400 for *Saccharomyces cerevisiae* [36].

Our species pan-genome for *Saccharomyces cerevisiae* was constructed using genomic data from 100 strains, 99 of which were previously included in the 100GS resource (Table S1) [50]. The resource includes 7 *Saccharomyces cerevisiae* genomes sequenced prior to 2015 and 93 *Saccharomyces cerevisiae* genomes sequenced *de novo* by the 100GS authors, taken from diverse genotypic and phenotypic backgrounds (populations referred to henceforth are as assigned by the 100GS authors after Liti *et al.* [50, 83]). The resource covers strains from laboratory, biotech, clinical and wild populations, which makes it an excellent dataset for carrying out *Saccharomyces cerevisiae* population genomics and pan-genomics studies of this kind. In their analysis, the 100GS authors screened *Saccharomyces cerevisiae* strains for aneuploidy, introgressed genes, phenotypically relevant single-nucleotide polymorphisms and non-reference genomic content [50]. The 100GS authors also assessed levels of resistance to environmental stresses such as sulphite and copper resistance, as well as fungicides such as ketoconazole [50].

A more recent study of 1011 *Saccharomyces cerevisiae* genomes included an analysis of the pan-genome of *Saccharomyces cerevisiae* in which the authors of that study detected non-reference genomic content by aligning strain genomes to the S288C genome using blastn, and extracting and annotating unique non-reference genes using an integrative multi-method procedure [36, 84]. Notably, despite a tenfold difference in the number of input strains, and different methods of identifying core and accessory genome content, both their study (4940 core genes) and our own (4900 core gene models) predict a similar-sized core *Saccharomyces cerevisiae* genome [36]. The 1011 genome study predicted an almost identical accessory genome to our analysis also; they identified 2856 accessory genes with varying distribution across 1011 genomes [36], whereas we identified an accessory genome of 2850 genes for our pan-genome dataset. The 1011 genome study also observed a number of evolutionary and functional trends within the *Saccharomyces cerevisiae* accessory genome; accessory genes were clustered within the subterminal regions of *Saccharomyces cerevisiae* genomes and some accessory genes may have originated via HGT from divergent yeast species or other fungi [36]. We observe similar trends in our analysis of the *Saccharomyces cerevisiae* accessory genome.

For the remaining three species, we constructed species pan-genome datasets based on strain genome assemblies that were available from GenBank at the start of our analyses. For each of these datasets, we attempted to sample strain genomes with as many diverse characteristics (e.g. geographical location, phenotype) as was possible with the genome assembly data available. Although there are a smaller number of strains sampled for these species pan-genomes, the sizes of these species’ core and accessory genomes are in line with our analysis of *Saccharomyces cerevisiae*, as well as larger analyses of species pan-genomes in fungi and other taxa. The *Candida albicans* species pan-genome dataset was constructed using data from 34 strains, predominantly clinical in origin, including both homozygous and heterozygous *MTL* mating-type strains (Table S1) [85]. A substantial amount of genome assembly data available for *Candida albicans* comes from strains isolated in hospitals; of the 34 strains in our dataset, 14 strains were clinical isolates from the USA alone (Table S1). A number of other strains were isolated from European and Middle East sources, but for 13 strains no information was available on the isolate source for the genome from GenBank. Perhaps as a consequence of a lower degree of environmental diversity due to sampling primarily clinical strains, the *Candida albicans* pan-genome has the smallest proportion of accessory gene content of the four species analysed in this study (~9 % of the entire species pan-genome). The *Candida albicans* pan-genome also has the lowest degree of variation in accessory genome size between individual strains of the four species analysed (Figs 3 and 6b). The UpSet distribution of the *Candida albicans* accessory genome illustrates this lower degree of variability within the *Candida albicans* pan-genome, as the most frequent sets are either singleton clusters or clusters that are missing an orthologue from one strain (Fig. S4). Despite this caveat, however, the *Candida albicans* pan-genome otherwise exhibits many of the same functional and evolutionary trends seen in the other three species we have investigated (as detailed below). With a broader sampling of strains found outside of a clinical context, a more accurate picture of the size of the *Candida albicans* accessory genome will be attained.

In contrast to *Candida albicans*, both our *Cryptococcus neoformans* var. *grubii* and *Aspergillus fumigatus* pan-genome datasets were constructed using a diverse array of strain genomes taken from both clinical and wild environments. The *Cryptococcus neoformans* var. *grubii* pan-genome dataset was constructed using clinical strain genomes isolated predominantly from human immunodeficiency virus positive patients from the USA and Botswana and wild-type strains sampled from Southern Africa sources (Table S1). *Cryptococcus neoformans* var. *grubii* has the largest proportion of accessory genes of the four species analysed (~20 % of the entire species pan-genome). As *Cryptococcus neoformans* is an intracellular pathogen in humans, it has to adapt to extreme variations in environmental stresses in order to survive. This is thought to lead to the high level of genomic rearrangement and instability seen in *Cryptococcus neoformans* [86]. It is possible that this in turn creates more novel genetic content, which may explain the higher level of accessory genome content seen in *Cryptococcus neoformans* var. *grubii*. Genomic instability as a result of pathogenic lifestyle fuelling pan-genome evolution has previously been observed in the wheat pathogen *Z. tritici* [37]. The *Aspergillus fumigatus* pan-genome dataset was constructed using 12 strain genomes sampled from clinical environments in the UK, USA and Canada, wild-type samples taken from China and from South American forest floors, and 2 strains isolated from surfaces within the International Space Station [77] (Table S1). Approximately 15 % of the *Aspergillus fumigatus* pan-genome is made up of accessory gene content, which is predominantly clustered in the subterminal regions of chromosomes (discussed below). There is a greater degree of variation in the accessory genome sizes of individual *Aspergillus fumigatus* strains than in the other species analysed, we believe that this is primarily an artefact of the smaller number of genomes in our dataset (at the time of writing our *Aspergillus fumigatus* dataset included almost all strain genomes available as assembly data on GenBank).

### Broad trends across fungal pan-genomes

#### Fungal core and accessory genomes enriched for potential infection and survival processes

Between 65 and 81 % of gene models per species pan-genome had at least one Pfam domain, while the proportion of gene models with GO data was between 42 and 54 % per species (Table 1). This variation is primarily down to a lack of human annotation for some species, and for *Cryptococcus neoformans var. grubii* in particular the lack of a dedicated GO-slim dataset. This can be seen in our statistical analyses of the distribution of GO terms in individual species pan-genomes; *Saccharomyces cerevisiae* currently has a far more detailed array of ontological terms than *Aspergillus fumigatus* for example (Table S2). In spite of gaps in ontological data for some of our species of interest, there are a number of patterns we can observe across multiple species in our GO analyses of fungal core and accessory genomes, as well as unique patterns of enrichment in some species. Many housekeeping terms such as translation, nucleic acid metabolism and oligopeptide metabolism are statistically over-represented in each fungal core genome we have analysed (*P*<0.05) (Table S2). There is an over-representation of similar cellular component terms in each of the three ‘yeast’ core genomes (i.e. all excluding *Aspergillus fumigatus*) (Table S2). This may reflect the morphological distinctions between these three species and *Aspergillus fumigatus*; however, the lack of dedicated annotation data for *Cryptococcus neoformans* var. *grubii* makes a definitive observation difficult. Terms relating to transport, localization and CAZY processes are statistically over-represented in fungal accessory genomes (Table S2). In part this is to be expected, as many fungi have varying numbers of copies of genes involved in CAZY and transport processes [87]. Terms relating to housekeeping processes are statistically under-represented in fungal accessory genomes, which may be due to potential gene dosage effects. The similar patterns of statistical over-representation for terms relating to intracellular membrane-bound organelles in the accessory genomes of *Cryptococcus neoformans* var. *grubii* and *Aspergillus fumigatus* may reflect infection or in-host survival processes for both pathogenic species (Table S2). Both the *Candida albicans* core and accessory species genome share similarly over-represented terms to their *Saccharomyces cerevisiae* counterparts, a reflection of the two species’ relatively close evolutionary relationship (Table S2).

Many of the terms that are over-represented in the *Cryptococcus neoformans* var. *grubii* core genome may reflect the species’ lifestyle as an intracellular pathogen (Table S2). Such terms include regulation of homeostasis and biological quality (e.g. cell mass), which are vital for *Cryptococcus neoformans* var. *grubii* to survive the plethora of environmental stresses it encounters in the host. Similarly, UPR is an over-represented molecular function in the *Cryptococcus neoformans* var. *grubii* core genome; the UPR pathway is known to influence thermoregulation in *Cryptococcus neoformans* var. *grubii* particularly during the initial infection period [88]. Another over-represented term in the *Cryptococcus neoformans* var. *grubii* core genome is signal transduction; many signal transduction pathways in *Cryptococcus neoformans* var. *grubii* play an important role in cell differentiation as well as pathogenicity (Table S2) [89]. The core *Aspergillus fumigatus* genome is enriched for small molecule biosynthesis and other biosynthetic processes, which concurs with previous comparative studies of *Aspergillus* species [90, 91] (Table S2). This also appears to agree with our findings of BGC conservation within the *Aspergillus fumigatus* species pan-genome (Table S5). Both transport and localization processes are over-represented within the *Aspergillus fumigatus* accessory genome, which may have an indirect role in the infection processes of *Aspergillus fumigatus. Aspergillus fumigatus* strain pathogenesis may, therefore, be influenced by accessory genome evolution, particularly within subterminal regions [92].

#### The fungal core genome is more ancient in origin than the fungal accessory genome

Our statistical analysis of the ancestral history of each fungal species pan-genome found that gene models of eukaryotic origin are statistically over-represented within fungal accessory genomes, while gene models of prokaryotic origin are statistically over-represented in fungal core genomes (*P*<0.05) (Table S3). In other words, genes of prokaryotic origin appear to be more likely to be syntenically conserved and universally retained within these fungal species (Table S3). This appears consistent with the observation that prokaryote-derived genes in *Saccharomyces cerevisiae* are essential for survival [70]. However, it appears that the accessory genome contains more genes that arose at some point during the evolution of eukaryotes and that may be more likely to be variably retained or lost within strains of fungal species (Table S3). This would concur with our analysis of the gains and losses of syntenic orthologues in fungal accessory genomes, which are largely mediated at the strain level.

#### HGT may only play a limited role in fungal pan-genome evolution

Given the extent of HGT in prokaryotes and its role in generating novel genetic content and in the evolution of prokaryotic gene families, it is likely that HGT plays a significant role in prokaryote pan-genome evolution. HGT in eukaryotes is known to be far less frequent than in prokaryotes however, so its impact on eukaryotic pan-genome evolution may be limited. We examined the extent of HGT into fungal accessory genomes from two potential sources of novel genetic content: prokaryote species and other species within the fungal kingdom. A screen for interdomain HGT events in each fungal accessory genome following previous methodology [71, 93] revealed low numbers of putative HGT events from prokaryote sources into fungal accessory genomes per species (Table S3). Gene transfer between prokaryotes and eukaryotes is a subject of some controversy, with different studies suggesting that interdomain HGT is alternately non-existent or a rare but real occurrence [25, 26, 94]. Regardless, from our analysis it appears that interdomain HGT is not an influencing factor on accessory genome evolution (and hence, pan-genome evolution) within fungi. We then applied a similar screen for HGT from other fungal species into fungal accessory genomes, and found that up to 8 % of fungal accessory genomes may be derived from intrakingdom HGT. There are caveats to consider when interpreting this finding however; although some of these events may be genuine incidences of HGT, it is equally plausible that these genes have undergone pseudogenization or have otherwise lost synteny in one or more strains/lineages. That the majority of potential donor species are close relatives in each analysis we performed may in part suggest this; for example, 96 of the 102 putative HGT events into the *Saccharomyces cerevisiae* accessory genome have a potential donor from the species in the same phylum (Saccharomycotina) and 379 of the 392 putative HGT events into the *Aspergillus fumigatus* accessory genome suggest transfer from other species in the subphylum Pezizomycotina (132 from *Penicillium* species alone) (Table S3). Although there appears to be greater evidence for intrakingdom HGT having a role to play in fungal accessory genome evolution than interdomain HGT, it is our opinion that a dedicated analysis of intrakingdom HGT in fungal accessory genomes using robust phylogenetic methods is required to test the true role of intrakingdom HGT in fungal pan-genome evolution.

#### Eukaryotic processes such as gene duplication may influence fungal pan-genome evolution

Between 29 and 41 % of fungal accessory genomes contain gene models which appear to be duplicates of core gene models that have undergone subsequent loss in some strains, possibly by pseudogenization, microsynteny loss or expansion in other strains (Tables 2 and S1). *Cryptococcus neoformans* var. *grubii* has the smallest proportion of these duplicated core gene models (and consequently, the highest proportion of accessory gene models that have potentially arisen via other processes) and *Aspergillus fumigatus* has the largest (Table S1). This accounts for between 3 and 7 % of the total size of fungal pan-genomes, with the smallest proportion in *Candida albicans* and the largest in *Aspergillus fumigatus* (Fig. 6, Table S1). These results appear to indicate that gene duplication, which is the driving factor of gene family expansion in eukaryotes, does play an important role in the evolution of fungal accessory genomes (and pan-genomes as a whole) [95, 96]. The larger proportion of duplicated core genes in *Aspergillus fumigatus* appears to reflect the greater extent of gene duplication and paralogue diversity within that species relative to *Cryptococcus neoformans* var. *grubii* and *Saccharomyces cerevisiae* [97]. Preliminary annotation of these gene models shows that many have putative or known functions in transport and outer membrane processes, which are processes that are often mediated by expanded gene families in fungi.

Mapping the presence or absence of syntenic orthologues within fungal accessory genomes finds that for each species the majority of syntenic orthologue loss events, through chromosomal rearrangement or gene loss, or the gain of new genes, has occurred within strains as opposed to more ancestral branches (Figs 2–5). We searched each set of singleton gene models from each reference genome against the reference protein set to assess the putative function(s) of some of these strain-unique genes. Many singleton gene models are homologous to membrane proteins, DNA/RNA-binding or transposition-related genes (e.g. *gag*/*pol* retrotransposons in *Saccharomyces cerevisiae*, DDE1 transposases in *Aspergillus fumigatus*), which are usually independently expanded or redistributed within individual fungal genomes [83, 98]. Between 30 and 60 % of singleton gene models within each species pan-genome dataset had at least one Pfam domain, a lower proportion than that seen in each species dataset (65–81 %) as a whole, which may be another artefact of gaps in human annotation (Table S2). Closely related strains of many species also appear to have similar accessory genome sizes (e.g. many clades within the *Saccharomyces cerevisiae* 100GS dataset, the reduced sizes of both *Cryptococcus neoformans* var. *grubii* C45 and MW-RSA852 relative to most other strains) (Figs 2–4). There is greater variation in the sizes of strain accessory genomes in *Aspergillus fumigatus*; however, this may be an artefact of taxon sampling (Fig. 5). *Saccharomyces cerevisiae* S288C itself had 31 singleton gene models not found in any other *Saccharomyces cerevisiae* strain. By comparison, the 100GS authors located 108 genes present in ≥1 strain but not in S288C and 28 genes unique to S288C [50]. In total, these analyses suggest that fungal pan-genomes evolve by innovations originating within fungi on the strain level, such as gene duplication or rearrangement, as opposed to being influenced by factors such as HGT from prokaryotic sources or larger species-level events.

#### Subterminal regions of fungal genomes may be harbours of accessory genome content

Analysis of the global distribution of core and accessory gene models shows that there is a statistically significant bias towards accessory gene models in the subterminal regions within three of the four reference genomes in our study and a statistically significant bias towards core gene models outside these subterminal regions in the same genomes (*P*<0.05) (Fig. S7a, c, d, Table S4). The sole exception is *Candida albicans* SC5314, wherein there is a statistically significant bias for core gene models within subterminal regions (*P*<0.05) (Fig. S7b, Table S4). The subterminal regions of chromosomes are usually areas of genomic instability in eukaryotes, so it is unsurprising that we observe greater breakdown of synteny in these regions [99]. Terminal and subterminal regions of chromosomes (i.e. telomeres and subtelomeric regions) are also known hotspots of recombination in fungi, which can lead to the evolution of novel genetic content, and in some fungi such recombinatory hotspots are potentially enriched for secreted proteins [100]. All fungal reference genomes possess at least one chromosome that is enriched for accessory gene models; these chromosomes may have undergone recombination or translocation events that lead to the breakdown of synteny or the eventual evolution of novel genes (Table S4). Such translocation events are known to have occurred within some strains of *Saccharomyces cerevisiae* and *Aspergillus fumigatus* in particular [86, 99, 101, 102]. In some reference genomes such as *Aspergillus fumigatus* Af293 large clusters of accessory genome content can be observed outside the subterminal regions, which may reflect instances of strain- or lineage-specific genomic rearrangement events (Fig. S7). Such rearrangements are linked to environmental adaptation and reproductive isolation in *Saccharomyces cerevisiae* genomes [103]. In *Cryptococcus neoformans* var. *grubii*, the greater degree of accessory genome content found outside subterminal regions may be a reflection of the role that genomic rearrangement plays in shaping the genomes of individual strains within the host [86].

#### Fungal core and accessory genomes encompass various biological pathways and phenotypes

Due to its position as arguably the most complete fungal model organism, there is a wealth of manually annotated functional data available for *Saccharomyces cerevisiae* that is lacking for other species. One such collection is the systematic mutation set available from the SGD, which includes amongst other datasets a systematically constructed genome-wide set of deletion phenotypes for many different strains of *Saccharomyces cerevisiae* [17, 73]. Using reciprocal blastp searches against the reference protein set as well as data from the systematic mutation set, we inferred the knockout viability of the core and accessory genomes of *Saccharomyces cerevisiae* S288C. We found that the core *Saccharomyces cerevisiae* S288C genome is not significantly over-represented for either knockout-viable or knockout-inviable genes (Table S5). This may reflect the fact less than 20 % of the genes encoded in the *Saccharomyces cerevisiae* S288C genome are thought to be essential for growth and, thus, likely knockout-inviable [104]. It is worth observing, however, that 962 of the 1031 predicted gene models with an inviable knockout phenotype are within the core *Saccharomyces cerevisiae* genome (Table S5). In contrast, there is a significant proportion of gene models within the *Saccharomyces cerevisiae* S288C accessory genome that are associated with a viable knockout phenotype (*P*<0.05), which appears to reinforce the more variable nature of species accessory genomes relative to core genomes (Table S5).

Unlike filamentous fungi such as *Aspergillus* species, many yeasts lack BGCs. Somewhat analogous to BGCs in *Saccharomyces cerevisiae* are small DP gene clusters of functionally related genes, which have been lost in other *Saccharomyces* and related species but were later regained in *Saccharomyces cerevisiae* via HGT or neofunctionalization [74]. Hall and Dietrich [74] previously described 14 such clusters, encompassing 38 reference and another 3 non-reference genes, which are involved in many different metabolic processes [74]. Our analysis of the distribution of 38 reference DP genes within the *Saccharomyces cerevisiae* pan-genome found one DP cluster that appears to be completely conserved in the pan-genome; a cluster on chromosome II containing three *GAL* genes that mediates the degradation of galactose to galactose-1-phosphate within the glycolysis pathway [105] (Table S5). Other clusters were highly conserved across almost all strains but not universally conserved in our dataset, i.e. a small number of strains. Such highly conserved clusters include two clusters involved in the metabolism of B vitamins; a three gene *BIO* biotin uptake cluster on chromosome XIV and a *SNO1-SNZ1* vitamin B6 metabolism cluster on chromosome XIII (Table S5) [74]. Another highly conserved six gene *DAL-DCG* cluster found on chromosome IX, the largest DP cluster, allows *Saccharomyces cerevisiae* to use allantoin as its sole nitrogen source through a pathway in which allantoin is converted to urea, which is then converted into ammonium by *DUR1-2* [106]. A *SAM4-SAM3* cluster that enables the usage of *S*-adenosylmethionine as a sulphur source has one of the two member genes missing in four strains (and is entirely absent in YJM969) (Table S5).

It is possible that some strains may simply be missing a syntenic orthologue of one or more genes in a cluster due to pseudogenization or synteny loss due to chromosomal rearrangement. Other DP clusters have more patchy distribution within the *Saccharomyces cerevisiae* species pan-genome, particularly those within subterminal regions in *Saccharomyces cerevisiae* S288C, which may indicate a greater breakdown of synteny or gene loss within these clusters. For some clusters, this may be due to functional redundancy; for example, three DP clusters are involved in vitamin B1 and B6 metabolism, the aforementioned *SNO1-SNZ1* cluster is conserved across almost all 100 strains whereas the other two clusters have patchier distribution or are totally missing in some strains (e.g. in the Indonesian strain YJM1244, two clusters are completely conserved but the other is absent) (Table S5). Other potential causes for this varying distribution of DP clusters may include environmental adaptations. One DP cluster that confers arsenic resistance is prevalent in many wine/European strains, but has much patchier conservation in non-European strains or strains with Malaysian or West African ancestry (such as SK1). One member gene of this cluster, *ARR3*, is absent in 49 out of the 100 strains in our dataset, including many mosaic strains with wine/European and Malaysian ancestry. Increased arsenic resistance has been observed in strains of European ancestry, likely as a result of anthropogenic influence on soil composition, which may explain the *ARR* cluster’s absence in some non-European strains [34, 76]. Additionally, the *ARR* cluster is located in the subterminal regions of chromosome XVI in *Saccharomyces cerevisiae* S228C; this suggests gene loss or chromosomal rearrangements amongst other events may be responsible for the absence of *ARR3* in the *ARR* cluster of many strains [34, 107].

Within the aspergilli and other fungi, functionally related genes involved in secondary metabolism pathways are often arranged into BGCs within the subterminal regions of chromosomes. These BGCs are involved in a range of infection and survival processes in the aspergilli, and subterminal regions themselves are believed to mediate the infection process of *Aspergillus fumigatus* in the human host [91, 92, 99, 108]. Our analysis of known BGCs in the *Aspergillus fumigatus* pan-genome found 14 BGCs that were completely conserved, a number of which are involved in the production of mycotoxins. Other BGCs have one or two syntenic orthologues that are missing in other strains, in these cases the majority of these genes may play more indirect roles in cluster function and, therefore, be less likely to be conserved within clusters, while some are only partially present or completely absent in some strains but are highly conserved in others (Table S5). An analysis of variation of *Aspergillus fumigatus* BGCs using short-read data by Lind *et al.* [109] found similar patterns of BGC variation to our gene-level functional analysis [109]. Lind *et al.* [109] observed some trends that explain the variation in BGCs within *Aspergillus fumigatus* in both their analysis and ours; for example, a fusarielin-like cluster we identified as missing from A1163 and partially present in other strains has gained pseudogenizing mutations in some strains but not others, whereas variation in other accessory BGCs is due to factors such as transposable elements (as is the case in a 27 member PKS cluster) or lineage-specific gene acquisition/loss events [109]. This suggests that some BGCs are invariably conserved due to the importance of their function (such as gliotoxins), while others may be lost in particular strains due to environmental adaptations or other factors.

### Other remarks

Compared to the increasing amount of software designed to construct and characterize bacterial and archaeal pan-genomes, little dedicated pan-genome software exists for eukaryote taxa. Our overall method of analysis, bespoke gene model prediction followed by pan-genome construction using PanOCT as the anchor method, is ad hoc but may point towards a sufficiently optimized syntenic method of pan-genome construction for eukaryotes in the future. On this point, it is worth noting that PanOCT’s current implementation has an exponential memory usage curve per genome added, which makes analysis of prokaryotic or eukaryotic datasets of this scale difficult without dedicated high-performance computational facilities [38]. The relative lack of GO information for some fungal species (e.g. *Cryptococcus neoformans* var. *grubii*, which currently lacks a dedicated GO-slim dataset) may have affected our functional characterization of fungal pan-genomes. We attempted to ameliorate this lack of data by using other sources of genomic information (e.g. knockout data from SGD for *Saccharomyces cerevisiae*), though their efficacy is ultimately dependent on human annotation. One caveat of large-scale pan-genome analysis of this kind may be the usage of genomes assembled via a reference-based approach as opposed to *de novo* approaches, which may then lead to an underestimation of accessory genome sizes within species pan-genomes due to underestimation of sequence diversity or inheritance of assembly artefacts from the reference genome [110]. The majority of genomes used for each species dataset were assembled using *de novo* approaches, for example, the 100GS dataset is predominantly *de novo* sequenced strains, so the potential effects of overreliance on reference-based assembly data may have been reduced in our study [50].

The size of a species pan-genome and its complements are ultimately dependent on the amount and the geographical or phenotypical variety of genomic data sampled. Methodological differences notwithstanding, our 100 strain analysis of the *Saccharomyces cerevisiae* pan-genome and the 1011 strain analysis by Peter *et al*. [36] predict similar-sized pan-genomes [36]. In contrast, our reconstruction of the *Candida albicans* pan-genome likely underestimates the true size of the *Candida albicans* accessory genome due to a lack of non-clinical genomic data. The greater variation of accessory genome sizes between individual strains of *Cryptococcus neoformans* var. *grubii* and *Aspergillus fumigatus* may be an artefact of there being fewer strain genomes available for both species, which would in turn affect the sizes of those species’ pan-genomes. There have been attempts to estimate the ‘true’ size of bacterial pan-genomes from existing data using different mathematical models, which vary from inferring almost infinite pan-genomes that increase in size with each strain added to stricter models that infer a more finite structure for most bacterial species [5, 6, 111]. Future analysis of fungal species pan-genomes should attempt to quantify their true size using similar methods.

### Conclusions

Evidence for the existence of pan-genomic structure has been demonstrated in eukaryotic taxa using a variety of methodologies. Using computational methods based on sequence similarity and conserved synteny between strains, we have constructed and characterized species pan-genomes for four model fungi: *Saccharomyces cerevisiae*, *Candida albicans*, *Cryptococcus neoformans* var. *grubii* and *Aspergillus fumigatus*. Defining core genomes as containing gene models syntenically conserved throughout species and accessory genomes as containing gene models of varying syntenic conservation and distribution throughout species, we find strong evidence for pan-genomic structure within fungi. Between 80 and 90 % of all potential gene models in fungal species are core gene models, with the remainder being accessory gene models that are strain-specific or specific to individual groups of strains. Fungal core genomes are enriched for genes of ancient origin and facilitate many essential metabolic, regulatory and survival processes in both commensal and pathogenic species. Fungal accessory genomes are enriched for genes of more recent origin, appear to evolve and vary in size by processes like gene duplication and gain/loss events within strains, and are enriched for genes involved in molecule transport and carbohydrate metabolism amongst other functions. Our analysis supports the growing amount of evidence for pan-genomic structure in eukaryotes.

## Data bibliography

1. McCarthy CGP, Fitzpatrick DA. Pipelines for eukaryotic pangenome analysis. Github: https://github.com/chmccarthy/pangenome-pipelines (2018).
