# Supplementary material for: Pan-genome analyses of model fungal species
Source: Microb Genom. 2019 Feb 4;5(2):e000243. doi: 10.1099/mgen.0.000243 (PMC6421352; doi:10.1099/mgen.0.000243)
Supplement: Supplementary File 1 [file mgen-5-243-s001.pdf]

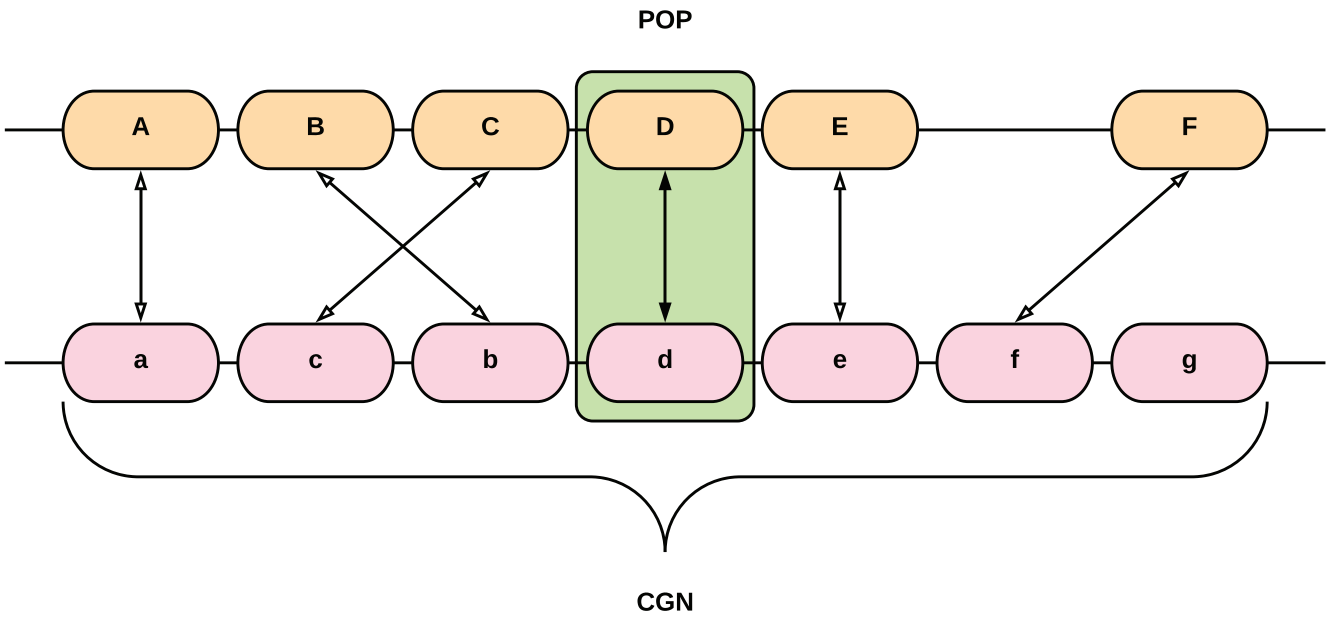

Figure S1. Simplified example of the methodology of PanOCT using two genomes (yellow and pink), adapted from [38]. Given a pair of potential orthologs (POP) from each genome, PanOCT will determine the extent of conservation of the surrounding genes (indicated by the white arrows) by calculating a "Conserved Genomic Neighbourhood" (CGN) score for the pair. PanOCT calculates this score for all possible POPs in a given dataset, and then merges the highest-scoring overlapping POPs together to form clusters of syntenically-conserved orthologs.

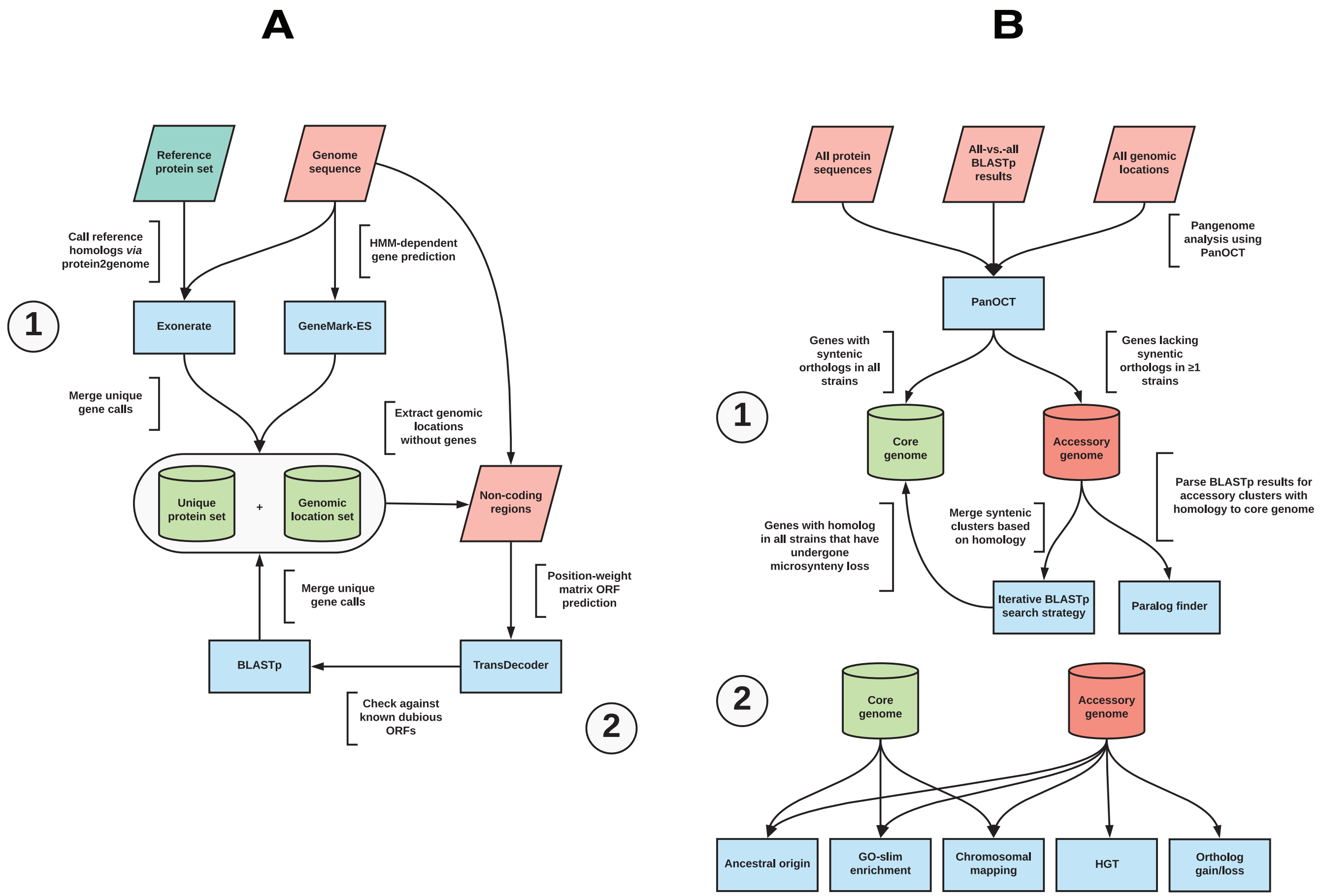

Figure S2. **A**: Workflow diagram of gene model sequence and location pipeline. Step 1 indicates the independent prediction of gene model sequences and locations using Exonerate and GeneMark-ES, while Step 2 indicates the prediction of putative coding gene models not found by either method in Step 1 using TransDecoder. **B**: Workflow diagram of pan-genome analysis using PanOCT and post-processing pipelines in Step 1, and various functional and statistical analysis of species core and accessory genomes in Step 2.

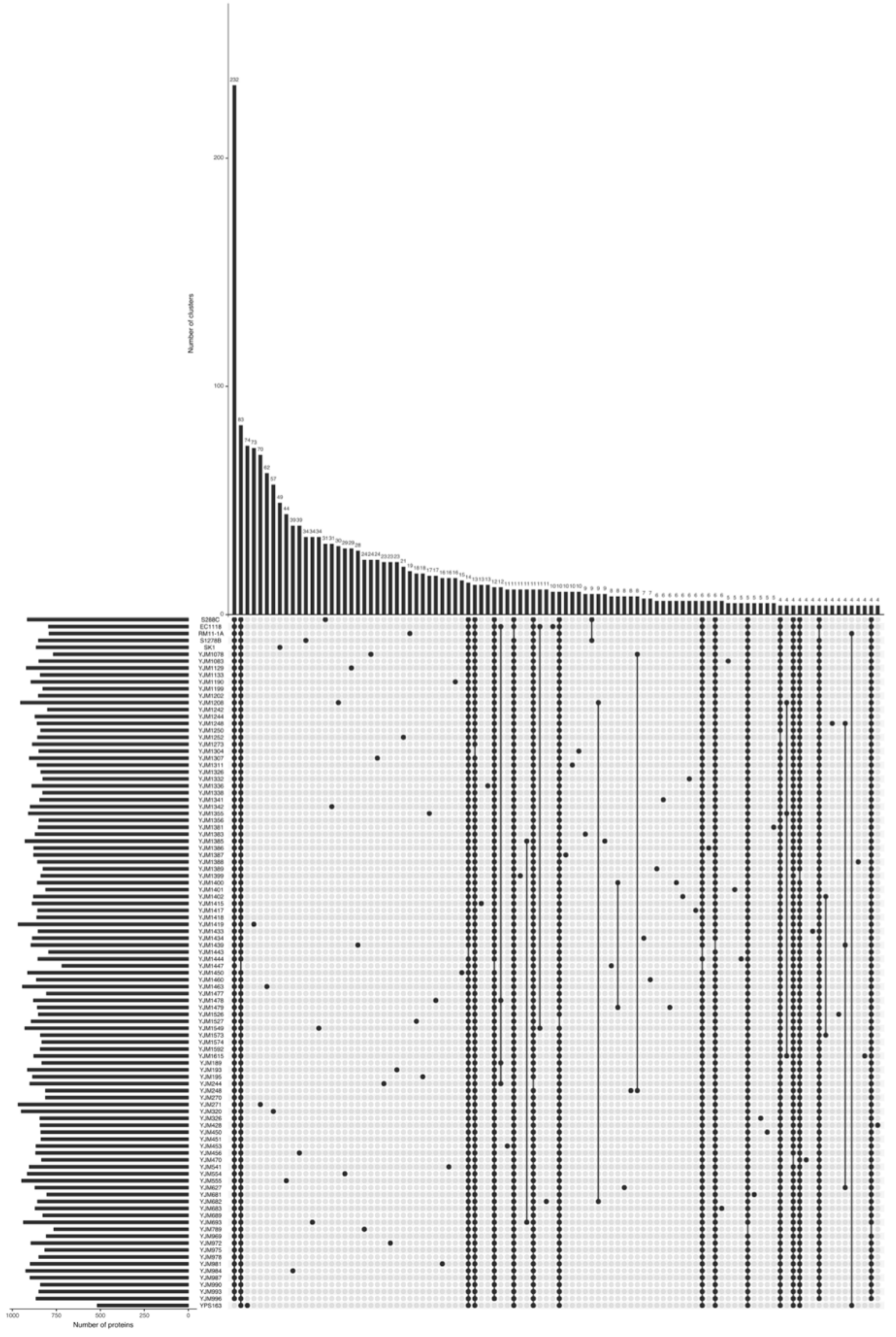

Figure S3. UpSet distribution of syntenic orthologs within the *Saccharomyces cerevisiae* pan-genome.

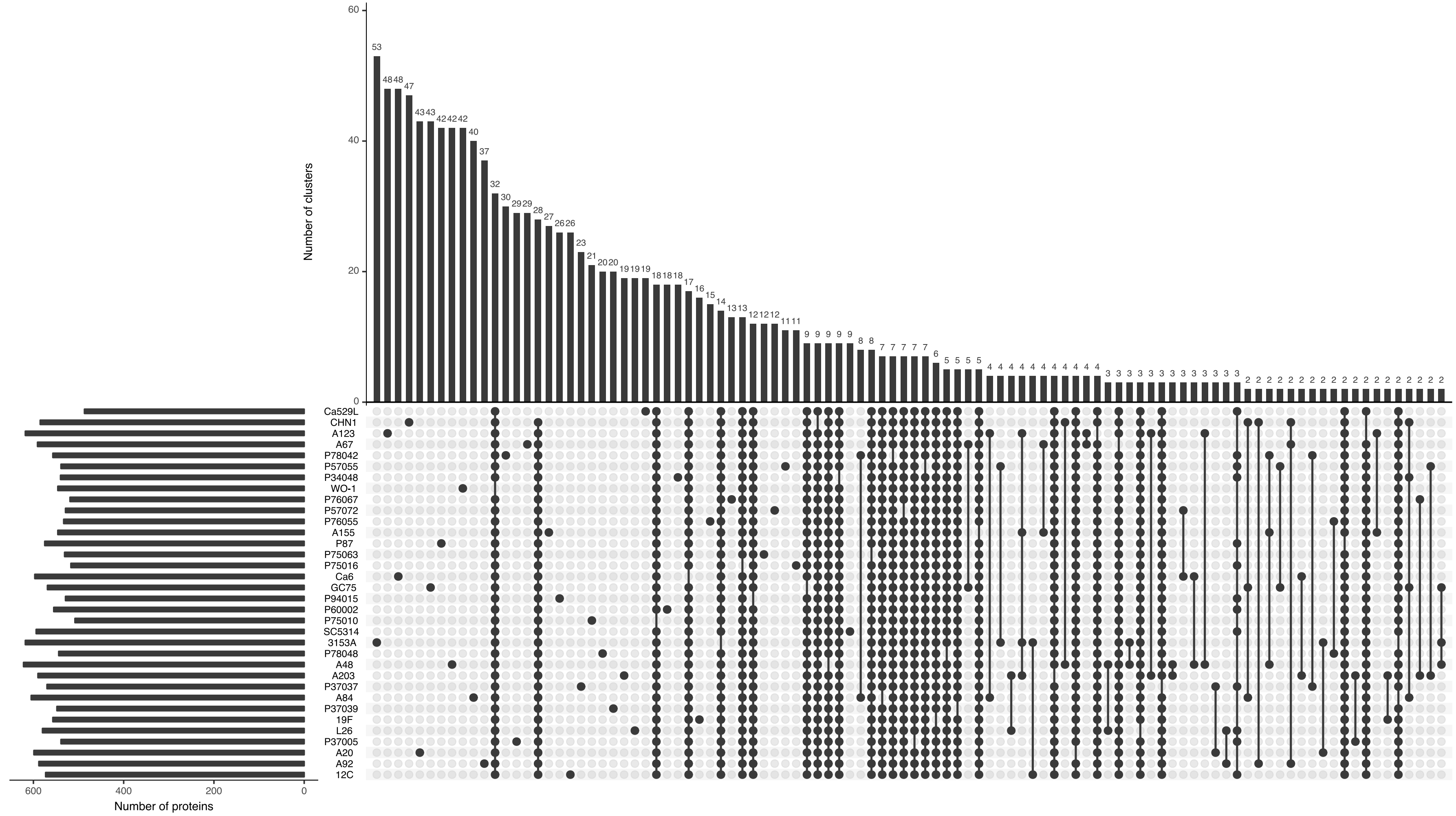

Figure S4. UpSet distribution of syntenic orthologs within the *Candida albicans* pan-genome.

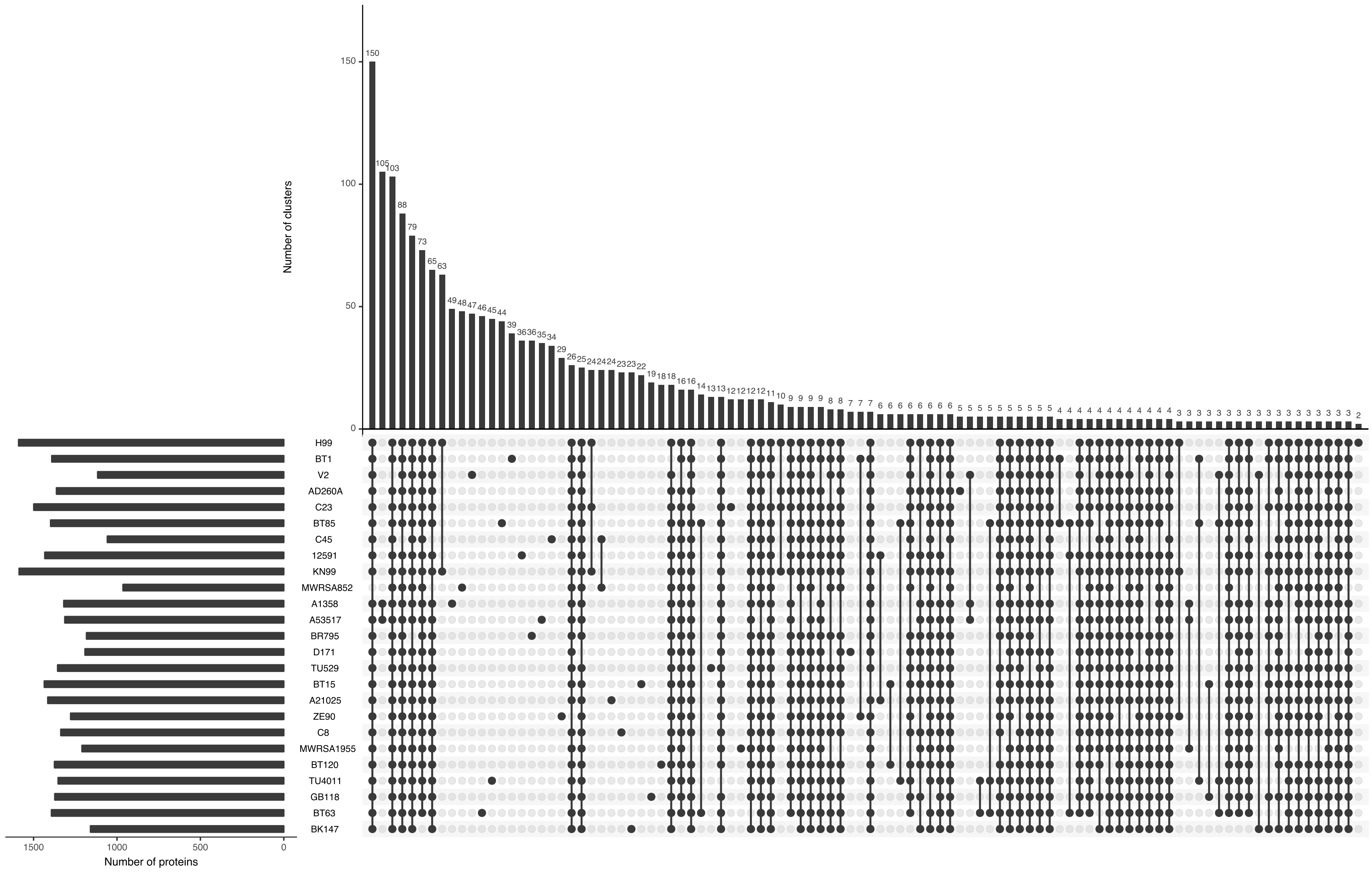

Figure S5. UpSet distribution of syntenic orthologs within the *Cryptococcus neoformans* var. *grubii* pan-genome.

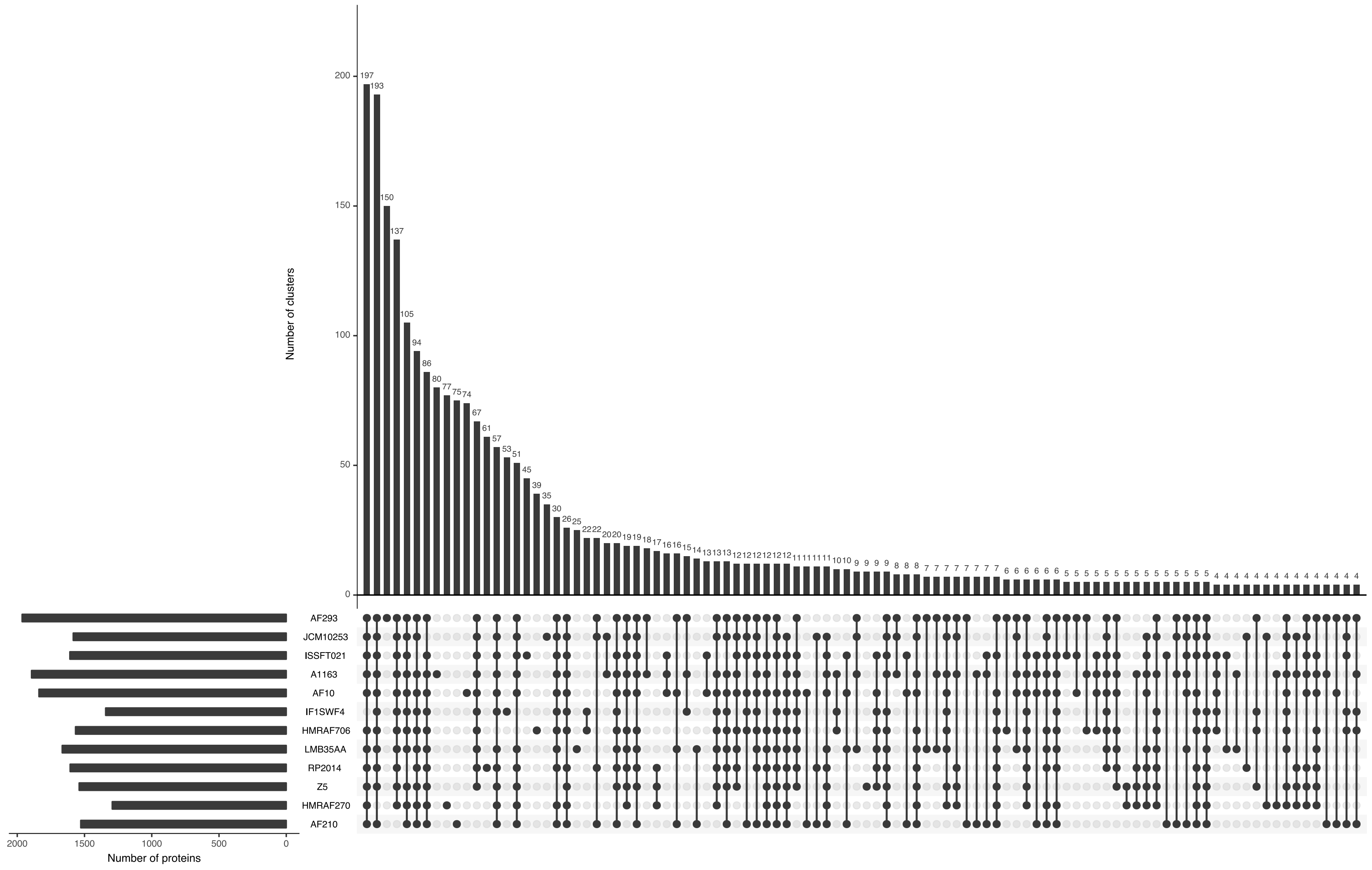

Figure S6. UpSet distribution of syntenic orthologs within the *Aspergillus fumigatus* pan-genome.

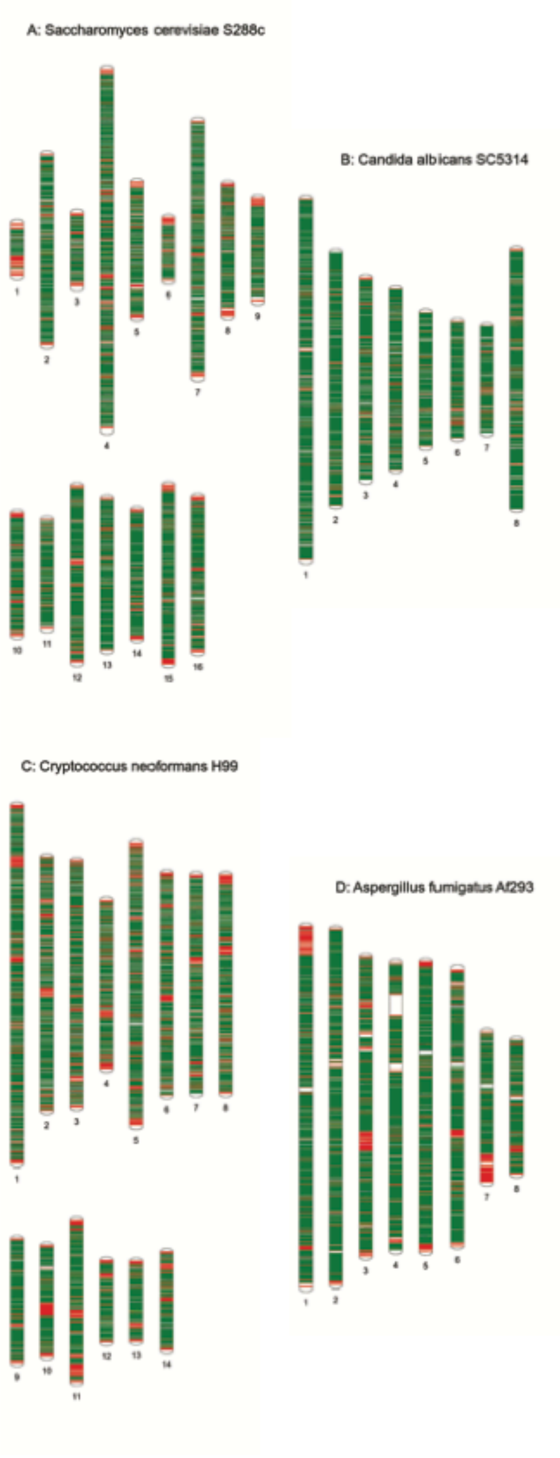

Figure S7. Plots of chromosomal locations of gene models along four fungal reference genomes. **A**: *Saccharomyces cerevisiae*, **B**: *Candida albicans*, **C**: *Cryptococcus neoformans* var. *grubii*, **D**: *Aspergillus fumigatus*. Reference strain identifiers provided in figure. Coloured bands correspond to predicted genomic locations of gene models. Green bands: core gene models, red bands: accessory gene models. Refer to **Table S4** for chromosomal distribution analysis.
